# Spatially-resolved pharmaco-ecology of immunotherapy resistance in lung adenocarcinoma: Single-cell and spatial transcriptomics for mechanism-matched combination therapies

**DOI:** 10.1016/j.tranon.2026.102994

**Published:** 2026-08-27

**Authors:** Yiming Hua, Zhanyang Luo, Zhimei Huang

**Affiliations:** a Department of Pharmacy, Taizhou Second People's Hospital Affiliated to Yangzhou University, Taizhou, Jiangsu, China; b Department of Pharmacy, Shanghai Pudong Hospital, Fudan University Pudong Medical Center, Shanghai, China

**Keywords:** Lung adenocarcinoma, Non-small cell lung cancer, Immunotherapy resistance, Single-cell omics, Spatial omics, Tumor immune microenvironment

## Abstract

Immune checkpoint inhibitors have reshaped the treatment landscape of lung adenocarcinoma, but primary, adaptive and acquired resistance are still the main clinical obstacles. This review integrates LUAD-specific evidence with broader NSCLC single-cell and spatial-omics studies, emphasizing lung adenocarcinoma where subtype-specific data are available. We discuss how therapeutic stress can select or induce malignant epithelial states that manifest as impaired antigen presentation, altered interferon signaling, partial epithelial-mesenchymal transition, stress tolerance, and hypoxia-related metabolic adaptations. These programs interact with dendritic cells, exhausted and precursor T cells, regulatory T cells, B cells, tertiary lymphoid structures, myeloid cells, cancer-associated fibroblasts, extracellular matrix, and blood vessels to generate a unique resistance-associated spatial immune niche. Special emphasis is given to the niche with adaptive suppression, immunologically excluded interstitial boundaries, myeloid-rich hypoxic-metabolic suppressive niches, antigen-presentation-low residual niches, and tertiary lymphoid structure (TLS)-associated immuno-tissue niche. We further highlight the translational potential of integrating single-cell and spatial features for biomarker discovery, prognostic assessment, interpretation of residual disease, and mechanism-matched combination therapies. Finally, we outline key challenges for clinical implementation, including sampling bias, platform heterogeneity, distinguishing association from causation, and the need for FFPE-compatible testing and prospective validation. Spatially informed single-cell analysis may enable lung adenocarcinoma to shift from descriptive immunophenotyping to ecological diagnosis and precision immune regulation.

## Highlights

• Immunotherapy resistance in lung adenocarcinoma arises from dynamic interplay among malignant cell plasticity, immune-stromal remodeling, and spatially organized niches.
• Single-cell and spatial transcriptomics define five resistance-associated niches: T-cell inflamed with adaptive suppression, immune-excluded stromal boundaries, myeloid-rich hypoxic-metabolic zones, antigen-presentation-low residual foci, and TLS-associated organized areas.
• Malignant epithelial cells initiate resistance through altered antigen presentation, IFN/JAK-STAT signaling, partial EMT, and hypoxic-metabolic adaptation, which collectively reshape the microenvironment.
• Biomarker strategies should move beyond single markers (e.g., PD-L1, CD8 density) toward composite spatial-ecological signatures that reflect tissue architecture and functional barriers.
• Future clinical translation requires protein validation, functional perturbation, multi-region sampling, and prospective trials to convert spatial ecological insights into mechanism-matched combination therapies.

## Graphical abstract

**Figure:**
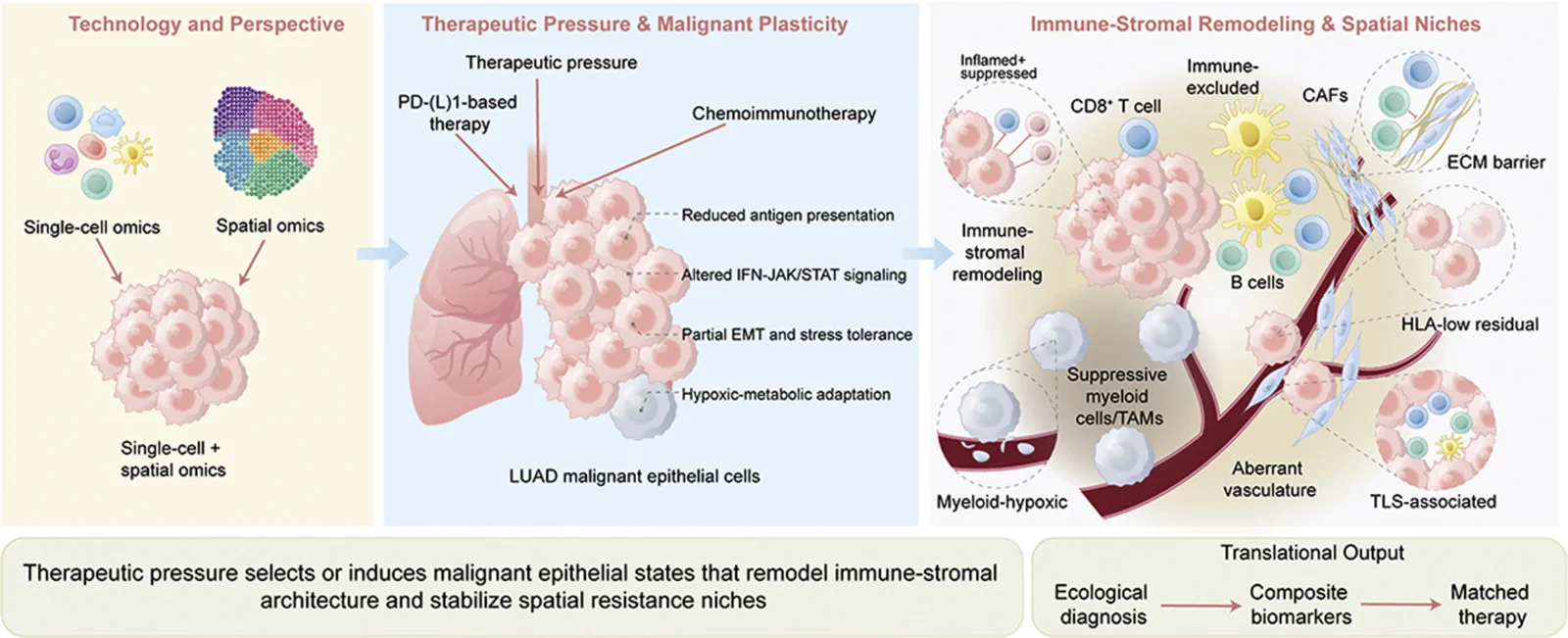

## Introduction: from lists of resistance mechanisms to a spatial ecological model

Lung adenocarcinoma is one of the most common pathological types of non-small cell lung cancer and is characterized by marked molecular heterogeneity [1,2]. PD-1/PD-L1 inhibitors and their combination with chemotherapy, antiangiogenic therapy, CTLA-4 inhibitors, and perioperative comprehensive therapy have significantly changed the treatment landscape of lung adenocarcinoma [[3], [4], [5]]. Some patients with advanced disease can achieve long-term remission, and some patients with resectable disease can achieve major pathological response or even pathological complete response after neoadjuvant therapy [[6], [7], [8]]. At the same time, the benefits of immunotherapy are still highly uneven: some patients have rapid progression from the beginning of treatment, while others have recurrence or metastasis after a brief remission [[9], [10], [11]]. Residual lesions after perioperative treatment often show complex immune escape characteristics [12,13].

Traditional biomarkers provide a necessary entry point for clinical decision-making, but their explanatory power remains limited. PD-L1 immunohistochemistry, tumor mutational burden, driver gene status, and conventional pathological indicators can reflect part of the immunogenicity or drug sensitivity background, yet they can hardly explain three recurring clinical phenomena: patients with high PD-L1 expression or relatively high TMB may still progress rapidly; the presence of CD8+ T cells within a tumor does not mean that these cells have penetrated tumor nests and mediated effective cytotoxicity; and different lesions in the same patient, or even different regions within the same lesion, may simultaneously display inflammation, exclusion, hypoxia, and myeloid suppression [[14], [15], [16]]. The key determinants of immunotherapy resistance often lie at the levels of local cell states, spatial contacts, and tissue architecture. These features are masked in averaged signals from bulk omics [17,18].

Single-cell sequencing and spatial omics provide a new level of resolution. Single-cell RNA sequencing can distinguish, at cellular resolution, the states of malignant epithelial cells, T cells, B cells, myeloid cells, fibroblasts, endothelial cells, and other microenvironmental populations [17,18]. TCR/BCR sequencing can track clonal expansion and local antigen responses [19]. Spatial transcriptomics, spatial proteomics, and multiplex imaging place these states back into tissue locations such as tumor nests, invasive fronts, stromal septa, perivascular regions, necrotic or hypoxic zones, and tertiary lymphoid structures (TLSs) [16,20]. The value of high-resolution technologies is not to mistake longer lists of cell subsets and markers for mechanistic explanation, but to build tissue ecological models that can explain the emergence, maintenance, and therapeutic targeting of resistance [21].

For an NSCLC framework with emphasis on LUAD, immunotherapy resistance can be viewed as a continuous process in which malignant cell-state transitions, immune-stromal feedback remodeling, and stabilization of spatial niches occur under therapeutic pressure, with LUAD-specific evidence used where available and mixed NSCLC evidence, including LUAD subsets where reported, treated as supportive context. The pre-treatment molecular background constitutes the initial immune ecology. Immunotherapy screens or induces altered antigen presentation, interferon response, EMT/stress, and metabolic programs in malignant epithelial cells [11,22,23]. These states collectively rewire T cells, DCs, B cells/TLS, myeloid cells, CAF, ECM, and blood vessels through secreted factors, ligand-receptor communication, and metabolism [17,24]. Finally, different spatial immune niches are formed, and the drug resistance manifests as primary, adaptive or acquired.

**Fig. 1:**
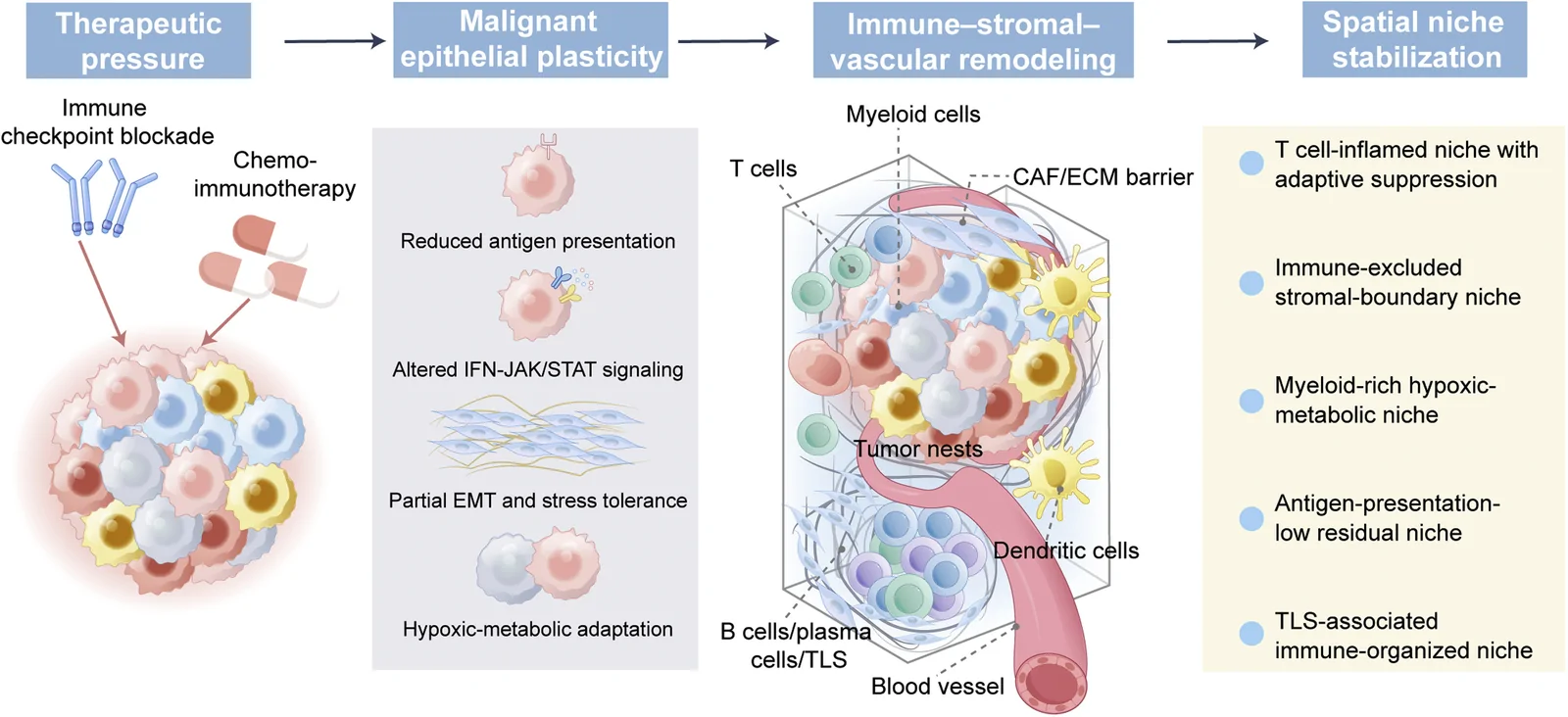
**A spatial ecological model of immunotherapy resistance in lung adenocarcinoma.** The figure illustrates a conceptual framework in which therapeutic pressure, including immune checkpoint blockade and chemoimmunotherapy, selects or induces plastic malignant epithelial states in lung adenocarcinoma. These states include reduced antigen presentation, altered IFN-JAK/STAT signaling, partial epithelial-mesenchymal transition and stress tolerance, and hypoxic-metabolic adaptation. Through secreted factors, ligand-receptor communication, metabolic competition, and matrix remodeling, malignant epithelial cells reshape T cells, dendritic cells, B cells/plasma cells and tertiary lymphoid structures, myeloid cells, cancer-associated fibroblasts, extracellular matrix, and vascular compartments. The remodeled tissue architecture can then stabilize several resistance-associated spatial niches, including T cell-inflamed niches with adaptive suppression, immune-excluded stromal-boundary niches, myeloid-rich hypoxic-metabolic niches, antigen-presentation-low residual niches, and TLS-associated immune-organized niches. Arrows indicate a conceptual and potentially iterative progression rather than a strictly linear sequence. Abbreviations: CAF, cancer-associated fibroblast; ECM, extracellular matrix; EMT, epithelial-mesenchymal transition; IFN, interferon; JAK/STAT, Janus kinase/signal transducer and activator of transcription; TLS, tertiary lymphoid structure.

Therefore, the subsequent discussion of this article will revolve around three levels: malignant cell plasticity, the spatial immune niche, and TLS maturation. Malignant cell plasticity emphasizes that malignant epithelial cells in lung adenocarcinoma are not in a fixed state, but under the combined influence of genetic background, epigenetic regulation, inflammatory signals, hypoxic metabolism, and therapeutic pressure, undergo reversible or semi-stable transitions between antigen presentation, interferon response, partial EMT, stress tolerance, metabolic adaptation, and residual cell states. The spatial immune niche emphasizes that these cell states do not exist in isolation, but together with specific immune cells, stromal cells, molecular programs, spatial boundaries, and cell communication form local tissue units with functional tendencies. Compared to the broad concept of tumor microenvironment, the spatial immune niche focuses more on how topological architecture influences immune cell entry, antigen recognition, local suppression, and therapeutic resistance. TLS maturation also needs to be understood at the spatial and functional levels, and should integrate B cell follicles, T cell zones, dendritic cells or follicular dendritic cells, high endothelial venule-like structures, BCR clonal maturation or plasma cell differentiation, as well as the spatial relationship between TLS and tumor antigen regions, rather than merely using CXCL13 or B cell marker expression as surrogate indicators. Taken together, these observations support a spatial ecological model in which immunotherapy resistance in lung adenocarcinoma emerges from the interaction between therapeutic pressure, malignant epithelial plasticity, immune-stromal-vascular remodeling, and the stabilization of distinct resistance-associated tissue niches. This conceptual framework organizes the following sections of the review (Fig. 1).

## Clinical resistance phenotypes and the molecular-ecological background: initial conditions for the resistance process

The clinical manifestations of immunotherapy resistance in NSCLC, particularly LUAD, include primary, adaptive, and acquired resistance [10,25]. Primary drug resistance usually refers to the lack of objective response or rapid progression after the initiation of treatment, suggesting that the tumor has been in the immune cold, immune-excluded, low antigen presentation, or myeloid/stromal suppression dominant state before treatment. Adaptive resistance occurs after an initial immune attack, and tumor cells and the microenvironment counteract immune pressure by upregulating alternative immune checkpoints, sustained interferon responses, myeloid cell recruitment, and matrix remodeling [22,26,27]. Acquired drug resistance often recurs or progresses after initial response. The core process of acquired drug resistance includes clonal replacement after immune editing, antigen loss, clonal function decline of T cells, residual cell expansion, and the formation of new spatial inhibitory niche [19,28,29]. These three classes of phenotypes are better viewed as cross-sections of the same evolutionary process at different temporal and spatial scales. Primary drug resistance emphasizes ecological limitation before treatment, adaptive drug resistance emphasizes feedback remodeling after immune challenge, and acquired drug resistance emphasizes residual clonal expansion and tissue ecology reconstruction after long-term immune editing. Oligoprogression and mixed responses further illustrate that different lesions in the same patient can be in different ecological states: some lesions form areas of inflammation that are accessible to T cells, and others are dominated by CAF/ECM barriers, myeloid suppression, or hypoxic metabolic structures [27,30,31].

The molecular background sets the initial boundaries for the above processes. EGFR-mutant and ALK-fused lung adenocarcinomas are often associated with a low mutation burden, a weak neoantigen burden, and a relative lack of an immune background for effective T-cell infiltration, so the benefit of immune checkpoint inhibitor (ICI) alone is usually limited [32,33]. KRAS/TP53 co-mutant tumors more often show inflammatory immune features, whereas KRAS/STK11 or KRAS/KEAP1 co-mutant tumors are more prone to low inflammation, myeloid enrichment, insufficient antigen presentation, oxidative stress, and enhanced metabolic adaptation [34,35]. TP53 mutation is associated with genomic instability and inflammatory signaling, but its immune consequences are jointly modulated by smoking exposure, co-mutation patterns, copy-number alterations, and the stage of tumor evolution [36,37].

STK11/KEAP1 provides one of the clearest examples of why genetic background needs to be interpreted ecologically. Skoulidis et al. reported in Cancer Discovery in 2018 that, in the 174-patient KRAS-mutant LUAD SU2C cohort, the objective response rate to PD-1 blockade was only 7.4% in the KRAS/STK11 co-mutant group, lower than the 35.7% observed in the KRAS/TP53 co-mutant group and the 28.6% observed in the KRAS-only group [38]. By integrating the CheckMate-057 cohort and mouse models, that study further supported an association between STK11/LKB1 loss and primary resistance to PD-1 axis blockade. Its strength lies in mutually supportive evidence from clinical cohorts, treatment outcomes, and model validation. Its limitation is that it primarily focused on KRAS-mutant LUAD and ICI monotherapy with PD-1/PD-L1 blockade. Ricciuti et al. further proposed in J Thorac Oncol in 2022 that the effect of STK11 and KEAP1 on PD-(L)1 efficacy is influenced by KRAS status, suggesting that these two genes should not be generalized as absolute negative biomarkers for all patients with LUAD [39]. In Nature in 2024, Skoulidis et al., using the POSEIDON study and immunocompetent models, showed that STK11 and/or KEAP1 alterations were associated with myeloid suppression and reduced CD8+ T cells, whereas dual immunotherapy plus chemotherapy incorporating CTLA-4 blockade could partially mitigate this resistant background [40]. Together, these three studies indicate that STK11/KEAP1 points to an ecological tendency characterized by myeloid skewing and T-cell restriction. Their differences in treatment regimen, KRAS context, and level of evidence mean that these alterations are better used for mechanistic stratification and combination-treatment design, rather than as a simple reason to exclude immunotherapy.

The relationship between driver gene status and immunotherapy response should therefore be understood probabilistically. Patients with EGFR mutations may still have PD-L1 up-regulation, treatment-induced inflammation, or local TLS. Patients with KRAS mutations may also be insensitive to ICI due to STK11/KEAP1 background, myeloid suppression, or CAF-mediated exclusion. What really translates the genetic background into a clinical resistance phenotype is the subsequent malignant cell state transition, immune/stromal remodeling, and spatial organization .Table 1

**Table 1:** Clinical resistance phenotypes and molecular-ecological backgrounds of LUAD/NSCLC immunotherapy resistance.

Entry point	Representative scenario	Dominant ecological tendency	Key cellular or molecular features to annotate	Spatial interpretation	Main resistance link	Key References
Primary resistance / rapid progression	No objective response or early progression after PD-1/PD-L1 blockade, despite standard clinical eligibility	Pre-existing immune-cold, immune-excluded, antigen-presentation-low, or myeloid/stromal-suppressed ecology	Low effective CD8+ T-cell infiltration; weak TCR clonal expansion; reduced HLA-I/APM signals; paucity of cross-presenting DCs; enrichment of suppressive TAMs, granulocytes, or CAF/ECM programs	Tumor nests show little productive T-cell contact, or T cells remain confined to stromal/perivascular regions	Baseline ecological non-permissiveness for ICI activity	[16,25,41]
Adaptive resistance after initial immune attack	Initial inflammatory response or partial control followed by counter-regulatory suppression	T-cell-inflamed but adaptively suppressed ecology	IFN-γ/CXCL9/10 activity; PD-L1, IDO1, TIM-3, LAG-3, TIGIT, CTLA-4-related signals; Treg enrichment; transition from effector or precursor-exhausted T cells toward terminal exhaustion	Tumor-T-cell contact may be present, but inhibitory programs concentrate at the tumor-immune interface	Adaptive resistance; response plateau; incomplete tumor clearance	[22,26,42]
Acquired resistance / relapse / oligoprogression	Progression after prior response or durable disease control; may appear as relapse, oligoprogression, or mixed response	Immune-edited residual clones with lesion-specific or region-specific suppressive niches	HLA-I/APM loss or downregulation; altered IFN signaling; neoantigen loss; residual malignant-cell states; dysfunctional tumor-reactive T-cell clones; hypoxic or stromal-protected residual foci	Resistant foci may localize to residual tumor islands, hypoxic/perivascular regions, stromal-protected regions, or separate metastatic lesions with different immune ecologies	Acquired resistance and recurrence; spatial explanation for mixed response	[10,11,29]
EGFR-mutant or ALK-rearranged LUAD	Oncogene-addicted LUAD considered for ICI monotherapy after targeted therapy or chemotherapy	Weakly immunogenic or immune-cold baseline ecology	EGFR sensitizing mutations or ALK fusion; generally low TMB/neoantigen burden; low CD8+ T-cell infiltration; PD-L1 expression may be dynamic and insufficient as a standalone indicator	Sparse tumor-reactive T-cell niches; immune cells may be peripheral, stromal, or bystander rather than productively intratumoral	Primary resistance tendency to PD-1/PD-L1 monotherapy; driver-positive disease should be interpreted separately	[32,33]
KRAS/TP53 co-mutant LUAD	Smoking-associated KRAS-mutant LUAD with TP53 co-mutation	More inflamed and IFN/checkpoint-enriched immune background, but still vulnerable to adaptive suppression	Higher TMB tendency; activated T-effector and IFN-γ signatures; PD-L1/checkpoint expression; CD8+ T-cell infiltration; preserved antigen-presentation context should be checked	More likely to contain T-cell-inflamed tumor nests or invasive-front immune infiltration; spatial tumor-T-cell contact and Tpex retention remain critical	Response-permissive initial condition rather than a guarantee of sensitivity; can evolve into adaptive or acquired resistance	[34,36]
KRAS/STK11 and/or KEAP1-altered LUAD	KRAS-mutant LUAD with STK11/LKB1 loss and/or KEAP1 alteration; sometimes also KRAS-wild-type KEAP1/STK11-altered contexts	Myeloid-rich, granulocytic, CD8-low, metabolically adapted, oxidative-stress-tolerant ecology	STK11/LKB1 loss; KEAP1/NFE2L2 pathway activation; reduced CD8+ T-cell infiltration; myeloid/neutrophil enrichment; impaired inflammatory or antigen-presenting tone; redox and metabolic adaptation	T-cell-poor tumor nests; myeloid-rich tumor cores or margins; hypoxic-metabolic regions; possible CAF/ECM reinforcement	Strong primary-resistance tendency, especially in KRAS-mutant LUAD, but not an absolute exclusion biomarker; treatment regimen and KRAS context matter	[23,[38], [39], [40]]
Antigen-presentation-low or IFN-JAK-altered malignant-cell state	Tumor cells reduce immune visibility before therapy or after immune editing	Immune-invisible or residual-tumor ecology	HLA-A/B/C, B2M, TAP1/2, PSMB8/9, NLRC5 downregulation or alteration; JAK1/2 or IFN-response defects; mismatch between CD8+ T-cell presence and tumor recognition	Residual tumor islands may be spatially close to immune cells but lack productive pMHC-I/TCR recognition; requires protein-level and genetic validation	Primary or acquired resistance; relapse despite apparent immune infiltration	[19,22,29,43]
EMT/TGF-β/CAF-ECM boundary phenotype	CD8+ T cells are present in tissue but fail to penetrate tumor nests	Immune-excluded stromal-boundary ecology	Partial EMT or invasive-front programs; TGF-β, CXCL12, AXL-related signals; ACTA2/TAGLN myCAF features; FAP, COL1A1, COL11A1, POSTN, LOX, MMP-related matrix remodeling	Thick CAF/ECM septa or tumor-stroma boundaries increase T-cell-to-tumor distance and reduce cytotoxic contact probability	Primary or adaptive resistance with a “pseudo-inflamed but excluded” phenotype	[15,27,44]
Myeloid-rich hypoxic-metabolic suppressive phenotype	Poorly perfused, necrotic, hypoxic, or metabolically stressed tumor regions; often overlaps with stromal remodeling	TAM/granulocyte-rich hypoxic-metabolic suppression	SPP1+ or TREM2/APOE-like TAMs; CXCL9:SPP1 macrophage polarity shift; S100A8/A9 or granulocytic features; HIF1A, CA9, VEGFA, LDHA, lactate/adenosine-related programs	TAMs and granulocytes localize to tumor cores, perinecrotic zones, perivascular niches, or ECM-rich borders where T-cell entry and function are impaired	Primary resistance, residual niche formation, and poor spatial immunotherapy signatures	[[45], [46], [47]]
TLS-/B-cell/plasma-cell-organized immune context	Mature TLS, plasma-cell signatures, or stem-immunity hubs are detected; some mTLS-positive tumors still fail ICI	Immune-organized and priming-capable ecology, but context dependent	Mature TLS; B-cell follicles; plasma cells; CXCL13/CCL19/CCL21; LAMP3+/CCR7+ DCs; TCF7+PD-1 + T cells; HEV-like structures; surrounding Treg, myeloid, or CAF barriers should be assessed	Functional interpretation depends on TLS maturity, distance to tumor antigen regions, CD8+ T-cell trafficking from TLS to tumor nests, and suppressive structures around TLSs	Usually response-permissive, but mTLS-positive primary resistance can occur when CAF/Treg/myeloid barriers prevent intratumoral killing	[[48], [49], [50], [51]]

**Note:** This table is intended as a conceptual guide rather than a deterministic biomarker checklist. The listed entry points are not mutually exclusive and include clinical resistance phenotypes, genomic backgrounds, malignant-cell states, and spatial immune niches. The same genomic alteration or cellular state may have different implications depending on treatment regimen, smoking background, co-mutation context, sampling time point, lesion site, and spatial organization. LUAD-specific evidence is prioritized where available; mixed NSCLC or pan-cancer evidence is used as supportive context. Gene and protein symbols follow standard nomenclature and are not expanded individually.

**Abbreviations**: APM, antigen-processing machinery; CAF, cancer-associated fibroblast; DC, dendritic cell; ECM, extracellular matrix; EMT, epithelial-mesenchymal transition; HEV, high endothelial venule; ICI, immune checkpoint inhibitor; LUAD, lung adenocarcinoma; mTLS, mature tertiary lymphoid structure; NSCLC, non-small cell lung cancer; pMHC-I, peptide-major histocompatibility complex class I; TAM, tumor-associated macrophage; TCR, T-cell receptor; TLS, tertiary lymphoid structure; Tpex, precursor/progenitor exhausted T cell; Treg, regulatory T cell.

## Malignant epithelial cell-state transitions: the starting point of the immune-escape ecology

The first layer of resistance to immunotherapy arises from the malignant epithelial cells themselves. Lung adenocarcinoma malignant cells continuously adjust their visibility, susceptibility to killing, and microenvironment shaping ability under immune stress, hypoxia, inflammatory factors, metabolic stress, and therapeutic exposure [21,52]. The great value of single-cell studies is their ability to localize these changes to the malignant cell itself, avoiding the misinterpretation of the bulk average signal as a state shared by all tumor cells.

Defective antigen presentation is a central way in which malignant cells reduce immune visibility. The recognition of tumor cells by CD8+ T cells depends on neoantigen processing, HLA-I presentation, and TCR recognition [19,53]. Decreased antigen processing and presentation programs such as HLA-A/B/C, B2M, TAP1/2, PSMB8/9 and NLRC5 will reduce the probability of tumor cells being recognized by cytotoxic T cells [29,54]. Single-cell sequencing can identify the state of antigen-presentation-low malignant cells, and further determine whether they are enriched in residual lesions after treatment, specific clones or immune-excluded regions. Such findings need to be interpreted with caution: decreased transcript levels are not equivalent to loss of HLA protein function, and low expression could also result from local hypoxia, low proliferation, or sample processing bias. A combination of HLA protein detection, mutation/copy number analysis, TCR clonal reactivity, and spatial proximity can be used to determine whether antigen presentation defects constitute a true driver of resistance [19,29].

IFN-γ/JAK-STAT signaling embodies the duality of the malignant cell state. Short-term IFN-γ signaling can enhance antigen presentation, chemokine expression and T cell recruitment, which is a component of effective anti-tumor immunity [55,56]. Persistent IFN signaling may coexist with adaptive resistance programs, including PD-L1, IDO1, and chronic ISG activation, whereas CXCL9/10 may reflect persistent inflammatory chemotaxis with functional consequences dependent on the spatial T cell access and inhibitory environment [22,45,57]. Importantly, IFN‑related signatures are often spatially heterogeneous within a single tumor. Regions with high IFN‑γ and CXCL9/10 expression may co‑exist with neighboring areas where hypoxia or stromal barriers suppress IFN responses, potentially contributing to mixed clinical responses or to the simultaneous presence of T‑cell‑inflamed and immune‑excluded niches in the same lesion. This spatial variability underscores that the functional consequence of IFN signaling must be interpreted together with local T‑cell access, antigen presentation integrity, and suppressive myeloid/CAF context, rather than as a uniform tumor‑wide property. Aberrant JAK1/JAK2 or downstream pathways can free tumor cells from IFN-mediated growth inhibition and enhanced antigen presentation [43,58]. IFN-high malignant cells at the single-cell level cannot be directly equivalent to treatment sensitivity, and should be explained by the integrity of antigen presentation, T cell contact, adaptive inhibitory signals such as PD-L1/IDO1, and myeloid/CAF structure.

EMT and partial EMT are manifestations of malignant cell plasticity at the levels of morphology, invasion, and tissue boundaries. Canonical EMT is associated with increased migration, invasion, and metastatic capacity [59,60]. Partial EMT retains some epithelial features while acquiring motility, resistance to apoptosis, and therapeutic tolerance [61,62]. EMT-like or partial EMT malignant cells are often located at invasive fronts, tumor-stroma interfaces, or hypoxic regions. Through TGF-β, AXL, ECM remodeling, and chemokine networks, they influence the positioning of CAFs, macrophages, and T cells [63]. Existing evidence consistently suggests that EMT programs are associated with immune exclusion, but the causal direction still needs to be separated [15,64]. Tumor cells may actively induce stromal barriers; CAFs and hypoxic environments may also induce EMT-like states in tumor cells [60].

Hypoxia and metabolic reprogramming provide malignant cells with another survival advantage under immune pressure. Deep tumor regions, perinecrotic areas, and poorly perfused zones are often enriched for HIF1A, VEGFA, glycolysis, lactate metabolism, adenosine pathways, and redox stress-adaptation programs [65,66]. These programs improve tumor-cell survival under low oxygen and nutrient competition, while altering the local immune ecology through abnormal vessels, myeloid-cell recruitment, and metabolic suppression of T cells [65,67,68]. A hypoxia score or glycolysis score itself is still a relevant indicator. It is closer to a mechanistic explanation when it is spatially co-localized with immune-cell exclusion, myeloid cell enrichment, low antigen presentation, or residual regions after treatment.

The external consequence of malignant cell state transition is to translate the intracellular stress program into microenvironment remodeling instructions. Signals such *As spp1*, VEGFA, TGFB1, CXCL12, CCL2, CSF1, MDK, and selected checkpoint-related molecules may connect malignant-cell stress programs with macrophages, neutrophils, CAFs, endothelial cells, and T cells [44,45,65]. SPP1-related programs have been repeatedly linked to myeloid suppression and ECM remodeling in multi-omics studies, but it is currently more suitable as a candidate communication axis and ecological stratification indicator [46,47]. To be elevated as a therapeutic target, it is necessary to clarify the main cell source, receptor cell, spatial location and functional perturbation results. Similarly, VEGFA or TGF-β expression can only be judged if it is a major driver or a concomitant phenomenon in a specific case by combining spatial boundaries, cell origin, and recipient cell status.

**Fig. 2:**
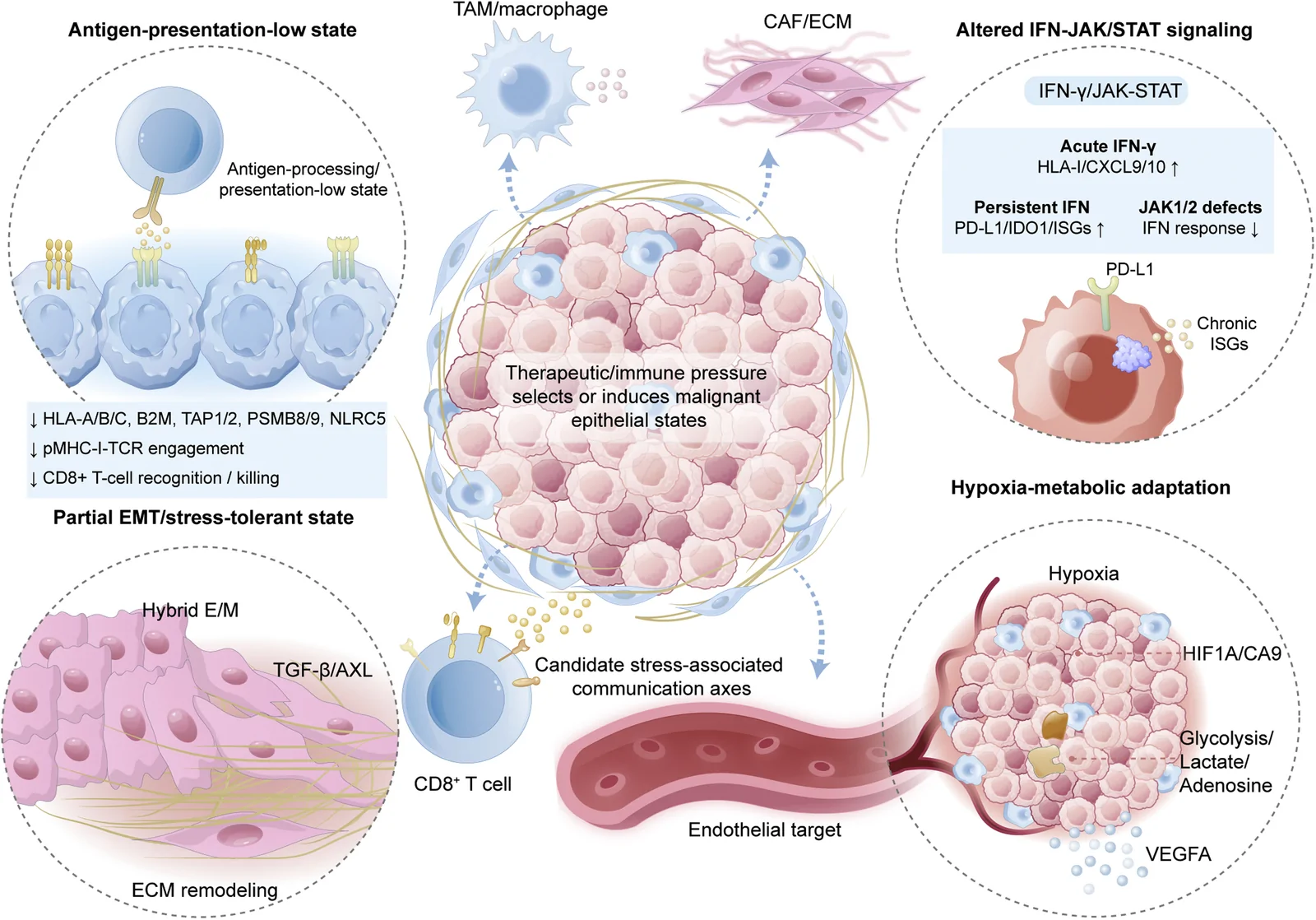
**Malignant epithelial cell-state transitions that seed immune escape under therapeutic pressure.** Therapeutic and immune pressure can select or induce distinct malignant epithelial states that reduce tumor-cell recognition and promote a resistance-permissive microenvironment. Antigen-presentation-low tumor cells show reduced expression of antigen-processing and presentation components, including HLA-A/B/C, B2M, TAP1/2, PSMB8/9, and NLRC5, thereby decreasing MHC-I-TCR engagement and CD8+ T-cell-mediated killing. Altered IFN-JAK/STAT signaling is context dependent: acute IFN-γ signaling may enhance HLA-I and CXCL9/10 expression, whereas persistent IFN signaling can coexist with PD-L1, IDO1, and chronic interferon-stimulated gene programs; JAK1/2 defects may further impair IFN responsiveness. Partial EMT and stress-tolerant states, often associated with TGF-β/AXL signaling and ECM remodeling, promote invasive behavior, stromal interaction, and immune exclusion. Hypoxic-metabolic adaptation, marked by HIF1A/CA9, glycolysis, lactate, adenosine, and VEGFA-related programs, supports tumor survival and favors suppressive myeloid and vascular remodeling. The central tumor nest and surrounding arrows highlight candidate communication axes linking malignant cells with TAMs/macrophages, CAF/ECM structures, CD8+ T cells, and endothelial cells. Abbreviations: AXL, AXL receptor tyrosine kinase; B2M, beta-2-microglobulin; CA9, carbonic anhydrase 9; CAF, cancer-associated fibroblast; CD8, cluster of differentiation 8; CXCL9/10, C-X-C motif chemokine ligand 9/10; ECM, extracellular matrix; EMT, epithelial-mesenchymal transition; E/M, epithelial/mesenchymal; HIF1A, hypoxia-inducible factor 1 subunit alpha; HLA, human leukocyte antigen; HLA-I, human leukocyte antigen class I; IDO1, indoleamine 2,3-dioxygenase 1; IFN, interferon; ISG, interferon-stimulated gene; JAK/STAT, Janus kinase/signal transducer and activator of transcription; MHC-I, major histocompatibility complex class I; NLRC5, NLR family CARD domain-containing 5; PD-L1, programmed death-ligand 1; pMHC-I, peptide-major histocompatibility complex class I; PSMB8/9, proteasome subunit beta 8/9; TAM, tumor-associated macrophage; TAP1/2, transporter associated with antigen processing 1/2; TCR, T-cell receptor; TGF-β, transforming growth factor beta; VEGFA, vascular endothelial growth factor A.

It follows that malignant cell plasticity constitutes the starting point of the causal chain: LUAD tumor cells reduce immune visibility, alter interferon responsiveness, acquire partial EMT or stress‑tolerant programs, and engage hypoxic‑metabolic adaptation, thereby generating the cellular signals that seed downstream immune and stromal remodeling (Fig. 2).

## Immune and stromal remodeling revealed by single-cell transcriptomics: from cell states to resistant communities

Malignant epithelial cell-state transitions must be received, amplified, and fixed by microenvironmental cells before they become sustainable immunotherapy resistance. Single-cell transcriptomics has moved this process from the level of “changes in cell proportions” to that of “cell-state reorganization” [16,18]. T cells can transition among progenitor exhausted, effector-like, terminal exhausted, and regulatory states [42,69]. DCs can display cross-presenting, mature migratory, or immunoregulatory states [70]. B cells can differentiate along naive/memory B-cell, germinal-center-like B-cell, and plasmablast/plasma-cell trajectories [49]. Myeloid cells can differentiate among CXCL9/10 inflammatory chemotactic states, SPP1 stromal-remodeling states, TREM2/APOE lipid-metabolic states, and neutrophil-associated states [45]. CAFs, the ECM, and endothelial cells jointly determine whether T cells can enter tumor nests [27].

Treatment-naive NSCLC atlases provide baseline references for understanding these states. For example, De Zuani et al. used single-cell and spatial transcriptomics across approximately 900,000 cells from 25 treatment-naive patients with NSCLC and showed complex myeloid reprogramming and spatial heterogeneity of immune cells in both adenocarcinoma and squamous cell carcinoma [16]. Because that study mainly analyzed treatment-naive samples, its evidence primarily reflects the baseline immune ecology of NSCLC rather than directly proving mechanisms of ICI resistance. Compared with treatment-naive samples, longitudinal or postoperative specimens collected after anti-PD-1-related neoadjuvant therapy more directly capture immune heterogeneity under therapeutic pressure. The large-scale single-cell atlas published by Liu/Zhang et al. in Cell in 2025 analyzed surgical samples from 234 patients with NSCLC after neoadjuvant anti-PD-1 plus chemotherapy or chemotherapy using scRNA-seq and scTCR-seq [71]. It identified post-treatment immune-ecological subtypes including TIME-NK, TIME-BE, TIME-Teff, TIME-Treg, and TIME-Mye (TIME means tumor immune microenvironment), providing more direct post-treatment evidence for interpreting MPR, non-MPR, and recurrence risk.

Immune and stromal remodeling in LUAD immunotherapy resistance can therefore be divided into four consecutive links: the success or failure of immune recognition and T-cell priming; weakening of T-cell effector function and fixation of exhaustion; formation of myeloid-stromal-vascular barriers; and stabilization of the post-treatment residual-lesion ecology. Single-cell transcriptomics defines these cell states. TCR/BCR sequencing tracks clonal expansion and antigen responses. Spatial omics further determines whether these states organize into tissue structures at tumor borders, stromal septa, perivascular regions, TLSs, or residual lesions.

### Success or failure of immune recognition and T-Cell priming

Effective antitumor immunity begins with antigen presentation and T-cell clonal priming. Antigen-processing and presentation programs in malignant cells, including HLA-I, B2M, TAP1/2, PSMB8/9, and NLRC5, determine the foundation of tumor-cell recognition. DC states determine whether antigens can be efficiently processed and presented to T cells. TLSs and B-cell responses determine whether local immune priming can form a more stable tissue structure. The value of single-cell transcriptomics lies in distinguishing cDC1, cDC2, LAMP3+ or CCR7+ mature/regulatory DCs and clarifying their functional relationships with T cells, B cells, and tumor regions. cDC1 mainly supports cross-presentation and CD8+ T-cell priming; cDC2 more often participates in CD4+ T-cell differentiation and local inflammatory regulation; and LAMP3+ mregDCs combine mature migratory, chemotactic, and immunoregulatory features, potentially connecting tumor antigen regions, TLSs, and T-cell priming zones [70,72].

TCR clonal analysis further converts the question from “whether T cells are infiltrating” to “whether tumor-reactive clones exist”. In a resectable NSCLC neoadjuvant anti-PD-1 cohort, Caushi et al. used MANAFEST to identify mutation-associated neoantigen-specific T cells and used their TCRs as “barcodes” to track single-cell transcriptional states [19]. That study found that both MANA-specific and virus-specific T cells were present within tumor tissue and that they carried distinct transcriptional programs. In non-MPR tumors, MANA-specific CD8+ T cells expressed higher levels of inhibitory molecules such as CTLA4, HAVCR2, and ENTPD1, whereas in MPR tumors, MANA-specific T cells were more biased toward memory/effector-related programs marked by IL7R, TCF7, and GZMK. These results indicate that the functional quality of tumor-reactive T cells is closer to the essence of therapeutic efficacy than total CD8+ T-cell density.

B cells and TLSs represent another key axis of immune priming, but their meaning should be upgraded from the simple presence or absence of TLSs to maturity, spatial location, and functional state. In a Nature Cancer study, Vanhersecke et al. proposed that mature TLSs were associated with improved objective response rate, progression-free survival (PFS), and overall survival (OS) after anti-PD-1/PD-L1 therapy, and that this predictive value was independent of PD-L1 expression and CD8+ T-cell density [48]. Patil et al. further found in an NSCLC atezolizumab cohort that an intratumoral plasma-cell signature predicted OS benefit from PD-L1 blockade, whereas the same signal did not predict benefit from chemotherapy alone [49]. The former study emphasizes the spatial organization of mature TLSs, whereas the latter emphasizes terminal B-cell differentiation and local humoral immune status. Together, they suggest that effective immune priming often depends on a local immune-reaction unit composed of T cells, B cells, DCs, and TLSs.

However, locally organized immune structures are not limited to classical mature TLSs. In a 2024 Nature Immunology study analyzing pretreatment NSCLC specimens before PD-1 blockade, Chen et al. described response-associated stem-immunity hubs [50]. These structures were enriched for TCF7+PD-1+CD8+ T cells, CCR7+LAMP3+ dendritic cells, CCL19+ fibroblasts, and related chemokines, and were clearly distinct from mature TLSs. This study suggests that spatially organized T cell-DC-fibroblast reaction units can represent another type of immune-priming ecology beyond TLSs. Conversely, Elfving et al. performed an H&E-level spatial assessment of TLSs in 680 resected NSCLC specimens and showed that TLSs are relatively common in NSCLC, that mature TLSs account for approximately 24% of evaluable cases, and that the number, location, and maturation status of TLSs are associated with local immune infiltration and survival [73]. Together, these studies show that the predictive value of the TLS/B-cell axis should not be reduced to single-marker expression of CXCL13 or B-cell markers. Instead, it should be interpreted together with maturity, plasma-cell differentiation, DC/Tfh-like cell support, distance to tumor nests, and surrounding suppressive structures.

### Weakening of T-Cell effector function and fixation of exhaustion

After T-cell priming, therapeutic efficacy depends on whether effector function can be maintained, expanded, and delivered into tumor nests. Single-cell studies have decomposed CD8+ T-cell exhaustion into a continuum [69,74]. Progenitor-like or precursor exhausted T cells associated with TCF7, IL7R, CCR7, LEF1, and GZMK retain higher proliferative and reactivation potential [42]. States associated with GZMB, PRF1, NKG7, GNLY, and IFNG reflect cytotoxic effector activity [69]. High expression of TOX, TOX2, HAVCR2, LAG3, TIGIT, ENTPD1, and PDCD1 suggests terminal exhaustion or suppressive fixation after chronic antigen stimulation [75,76]. CXCL13+ CD8+ T cells are often considered candidate tumor-reactive-like T cells in multiple cancers [77], but their functional meaning needs to be interpreted in relation to TCR clonal ex, spatial contact, and treatment outcome.

In Nature Cancer in 2022, Liu et al. performed longitudinal single-cell RNA sequencing and paired TCR sequencing on 47 tumor biopsies from 36 patients with NSCLC receiving PD-1-related therapy [42]. They found that responding tumors accumulated precursor exhausted T cells after treatment, whereas nonresponding tumors failed to accumulate this population. The study also proposed the concept of “clonal revival” in which effective T-cell responses after PD-1 blockade mainly arise from expansion of local precursor exhausted T cells and supplementation of tumors by peripheral emerging or pre-existing clones, rather than simple reversal of terminally exhausted cells. This conclusion is consistent with the study by Caushi et al. on neoantigen-specific T cells: whether immunotherapy works depends critically on whether there is a pool of tumor-reactive T cells that can expand, migrate, and enter tumor tissue [19].

Tregs and adaptive suppressive signals further determine whether effector T cells can complete tumor-cell killing. Treg states associated with FOXP3, IL2RA, CTLA4, IKZF2, TNFRSF18, ENTPD1, and CCR8 can weaken effector T cells through CTLA-4-mediated deprivation of costimulation, IL-10/TGF-β secretion, adenosine metabolism, and local nutrient competition [[78], [79], [80]]. When tumor cells, myeloid cells, and CAFs upregulate PD-L1, IDO1, LGALS9, CD155, NECTIN2, or the adenosine axis, an initially inflammatory response may be converted into suppressive feedback [22,26]. When Treg expansion, accumulation of terminally exhausted T cells, SPP1+ macrophages, and CAF/ECM barriers coexist, T-cell dysfunction more readily moves from reversible inhibition into a stable resistant state.

Neoadjuvant chemoimmunotherapy specimens provide a more direct window into T-cell fate and post-treatment immune heterogeneity. In the large-scale resource study published by Liu/Zhang et al. in Cell in 2025, surgical samples from 234 patients with NSCLC after neoadjuvant anti-PD-1 plus platinum-based chemotherapy or chemotherapy were analyzed using scRNA-seq and scTCR-seq [71]. Five post-treatment TIME subtypes were identified: TIME-NK, TIME-BE, TIME-Teff, TIME-Treg, and TIME-Mye. Patients with MPR were more often enriched for FGFBP2+ NK/NK-like T cells, memory B cells, or effector T cells. Patients with non-MPR more commonly showed enrichment of CCR8+ Tregs or insufficient clonal expansion of Tex-relevant T cells. The study also proposed that the abundance of progenitor exhausted T cells may serve as a candidate predictor of postoperative recurrence risk, suggesting that whether T cells remain in a reactivatable state may reflect residual immune control more effectively than pathologic response alone. These findings form a continuous chain of evidence with the clonal revival model proposed by Liu et al. in Nature Cancer in 2022 and with the work by Caushi et al. on neoantigen-specific T cells [42]. Whether immunotherapy is effective depends not only on the presence of CD8+ T cells, but also on whether tumor-reactive or Tex-relevant clones retain re-expansion potential, and whether they are suppressed by CCR8+ Tregs, terminal exhaustion programs, or myeloid/stromal barriers. From the perspective of spatial interactions, the small-sample single-cell and spatial transcriptomic study by Cui et al. in Molecular Cancer in 2025 further suggested that communication among SELENOP+ macrophages [81], antigen-presenting CAFs, and T cells may be associated with sensitivity, whereas the distributions of CD4+ Tregs and myofibroblastic CAFs are more consistent with a suppressive TME.

### Formation of myeloid-stromal-vascular barriers

Once T-cell effector activity is limited, the local tumor often enters a tissue-remodeling stage jointly dominated by myeloid cells, CAFs, the ECM, and blood vessels [16,27]. Single-cell transcriptomics shows that myeloid cells in NSCLC are highly heterogeneous [16,24]. CXCL9/10+ macrophages more closely resemble an IFN-induced inflammatory chemotactic state and may support T-cell entry [45]. SPP1+ macrophages are often associated with ECM remodeling, collagen deposition, CAF interaction, and T-cell exclusion [82]. TREM2+/APOE+ macrophages are often accompanied by lipid metabolism, phagocytic, and immunosuppressive programs [83]. FCN1+ and S100A8/A9+ monocytes and ARG1- or CXCR2-related neutrophil-like states may participate in inflammatory recruitment, tissue repair, and suppressive remodeling [16,83]. The traditional M1/M2 dichotomy cannot capture the continuous transitions among these states.

The relationship between myeloid-cell states and immunotherapy response should not be summarized by macrophage abundance. The key lies in polarity, spatial location, hypoxic background, and neighboring cellular networks. In Science in 2023, Bill et al. proposed a CXCL9^45^:SPP1 macrophage-polarity axis, showing that TAM polarity can be summarized by CXCL9 and SPP1 expression states and is associated with TME programs and clinical outcomes across cancers. That study also suggested that IFN-γ tends to drive a CXCL9-high inflammatory chemotactic state, whereas hypoxia more readily promotes *A. spp1*-high stromal-remodeling state. When this pan-cancer framework is placed back into NSCLC, its functional consequences should be interpreted together with lung cancer-specific spatial studies. Zhu et al. integrated NSCLC single-cell and spatial transcriptomic data in 2025 and found that TAMs are enriched in tumor cores and may form immunosuppressive interactions with T cells through ligand-receptor axes such *A. spp1*-CD44, NECTIN2-TIGIT, and HLA-E-CD8B [47]. Functional validation further suggested that macrophage-derived SPP1 can enhance expression of T cell exhaustion-related molecules. These findings provide additional evidence at the macrophage-T cell suppressive-interaction level for SPP1-related myeloid states. They also suggest that future therapeutic strategies should not focus only on PD-1/PD-L1, but should consider blocking suppressive communication between myeloid cells and T cells.

SPP1+ macrophage-CAF interaction is one of the most representative resistance modules identified in recent NSCLC spatial single-cell studies. In Nature Genetics in 2025, Yan et al. analyzed NSCLC samples collected before and after neoadjuvant ICB-chemotherapy using single-cell and spatial transcriptomics [82]. They found that tumor cells and SPP1+ macrophages can interact with COL11A1+ CAFs to promote collagen-fiber deposition and entanglement at tumor borders, forming a spatial barrier that impedes T-cell entry into tumor nests and is associated with poor prognosis. The value of this study lies in its simultaneous provision of pretreatment and post-treatment single-cell, spatial transcriptomic, and tissue-boundary evidence, which elevates the SPP1+ macrophage-COL11A1+ CAF axis from a routine marker combination to a resistance model with spatial structural meaning.

Other NSCLC studies support this model from different angles. In a 2025 integrated single-cell and spatial transcriptomic study of NSCLC, Xiao et al. observed that SPP1 in tumors is mainly expressed *By spp1* + macrophages, and that SPP1+ macrophages show spatial proximity and interaction with FAP+ fibroblasts [46]. Zhu et al. further suggested that SPP1-CD44 may also participate in macrophage-T cell immunosuppressive communication [47]. While SPP1 can also be expressed by malignant cells in some contexts, the resistance-associated SPP1 signal in LUAD is predominantly derived from macrophages, as supported by single-cell and spatial co-localization studies. However, the cell of origin may affect downstream effects; tumor-derived SPP1 might more directly promote autocrine survival and EMT, whereas macrophage-derived SPP1 primarily acts on fibroblasts and T cells via SPP1-CD44 and SPP1-integrin axes. Future targeting strategies should therefore consider the dominant source and spatial distribution of SPP1 rather than treating it as a single uniform target. In addition, Herzog et al. showed in NSCLC models that tumor-associated fibrosis can weaken T-cell immune surveillance and reduce the efficacy of immune checkpoint blockade, providing functional mechanistic support for the effect of CAF/ECM barriers on ICI response [84]. Taken together, SPP1+ myeloid cells and CAF/ECM barriers often converge on immune-excluded or myeloid-rich suppressive niches. However, whether SPP1 itself is a direct resistance driver, a readout of hypoxic/stromal states, or a druggable therapeutic target still requires further confirmation through in vivo perturbation, protein validation, and prospective stratification studies.

CAF states determine whether myeloid signals can be fixed into tissue architecture. Single-cell and spatial proteomic studies commonly classify CAFs into myCAF, iCAF, matrix-remodeling CAF, antigen-presenting-like CAF, IFN-responsive CAF, or tumor-like CAF states [44]. myCAFs are characterized by contractile and matrix-deposition programs such as ACTA2, TAGLN, MYL9, and COL1A1. iCAFs are enriched for inflammatory and chemokine factors such as IL6, CXCL12, CXCL14, and CCL2^82^. Matrix-remodeling CAFs commonly express COL11A1, POSTN, FAP, MMP11, and LOX and promote ECM remodeling and T-cell exclusion. Antigen-presenting-like CAFs express antigen-presentation-related molecules such as HLA-DRA and CD74, but their true immune function requires interpretation at the protein level and in functional experiments [81].

In Cancer Cell in 2024, Cords et al. used spatially resolved single-cell imaging mass cytometry to analyze 1070 NSCLC cases and stratified patients based on 11 CAF phenotypes and their spatial distributions [44]. Inflammatory CAFs and IFN-responsive CAFs were associated with immune infiltration and better survival, whereas matrix CAFs were associated with low immune infiltration and poorer survival, and tumor-like CAFs were significantly associated with adverse prognosis. This study demonstrates that the same “CAF-rich” phenotype may correspond to inflammatory support, antigen-presentation-like function, or stromal exclusion. The clinical meaning of CAFs depends on their specific phenotype and spatial location and cannot be reduced to a single suppressive cell population.

Endothelial cells and neutrophils further reinforce exclusionary tissue architecture. Abnormal angiogenesis can generate poor perfusion, hypoxia, and restricted immune-cell extravasation through programs involving VEGFA, KDR, FLT1, ANGPT2, and HIF1A [41]. By contrast, HEV-like endothelial cells may support TLS formation and lymphocyte entry. In Nature Genetics in 2025, Aung et al. integrated multi-cohort data from 234 patients with advanced NSCLC receiving PD-1-based immunotherapy and constructed predictive models using spatial proteomics and compartment-based spatial transcriptomics [41]. The spatial proteomic cohort included 67 cases, and the spatial compartment-based transcriptomic cohort included 131 cases. The study found that resistance-associated spatial cell-type signatures included proliferative tumor cells, granulocytes, and vascular-associated compartments, whereas response-associated signatures included macrophages and CD4 T cells. The cell-to-gene signature further predicted outcomes across multiple cohorts. These results suggest that vascular, granulocytic, and stromal compartments, together with tumor-cell proliferation, constitute a resistance-associated spatial background. Immunotherapy predictive models need to incorporate vascular, myeloid, and stromal compartments, rather than being limited to PD-L1 or CD8 density.

### Stabilization of the residual-lesion ecology and seeds of recurrence

Post-treatment residual lesions are a key setting in which to observe the formation of resistant communities. After immunotherapy or chemoimmunotherapy clears sensitive cells, residual regions are often enriched for malignant cells with low antigen presentation, EMT-like programs, hypoxic metabolism, cell-cycle arrest, stress tolerance, or stem-like programs [11]. They are accompanied by terminal exhaustion of T cells, Treg cells, SPP1^+^/TREM2^+^ myeloid cells, COL11A1+FAP+ CAFs, ECM barrier and abnormal vascular structure. At this point, drug resistance manifests as a tissue state maintained by residual malignant clones and protective microenvironment.

Paired or sequential analysis of pre-treatment biopsy and post-treatment surgical specimens helps to distinguish between pre-existing resistant seeds and treatment induction status [12,21]. For residual disease after neoadjuvant immunochemotherapy, the key question is not only the proportion of residual tumor cells but also whether reactivable immune control is retained in the residual region. The absence of progenitor exhausted T cell expansion, loss of tumor reactivity or Tex-relevant TCR clones, or the retention of dominant clones outside the stromal septum in the residual region after treatment indicate that immune pressure has not continued to enter the tumor nest. In the presence of memory B cells, plasma cells, mature or functional TLS, Tfh-like cells, BCR clonal maturation, and sustained expansion of tumor-reactive/Tex-relevant T cell clones, the residual lesions may still have immune responses that can be reactivated [48,49].

The large-cohort Cell study by Liu/Zhang et al. in 2025 provides post-treatment evidence for this interpretation [71]. In 234 surgical specimens from patients with NSCLC after neoadjuvant anti-PD-1-related therapy, the study identified distinct TIME subtypes and showed that patients with non-MPR are not a single resistant group. Some patients had relatively high levels of both Tex-relevant T-cell clones and CCR8+ Treg clones, suggesting coexistence of immune activation and immune suppression. Other patients showed insufficient expansion of Tex-relevant clones, suggesting inadequate initial immune activation. The study also found that progenitor exhausted T cells were associated with recurrence-free survival, indicating that Tpex abundance may serve as a candidate indicator of postoperative recurrence risk and residual immune-control capacity. Residual lesions therefore should not be viewed simply as tumors left behind after immunotherapy failure. Rather, they should be considered recurrence niches selected by malignant clones, T-cell clonal fate, Treg suppression, myeloid/CAF barriers, and local immune-organizing structures. Residual-lesion studies should also incorporate the fate of TCR/BCR clones. The clonal revival model proposed by Liu et al. and the plasma-cell predictive model proposed by Patil et al. support this interpretation from the complementary perspectives of T-cell clonal expansion and terminal B/plasma-cell differentiation [42,49].

**Fig. 3:**
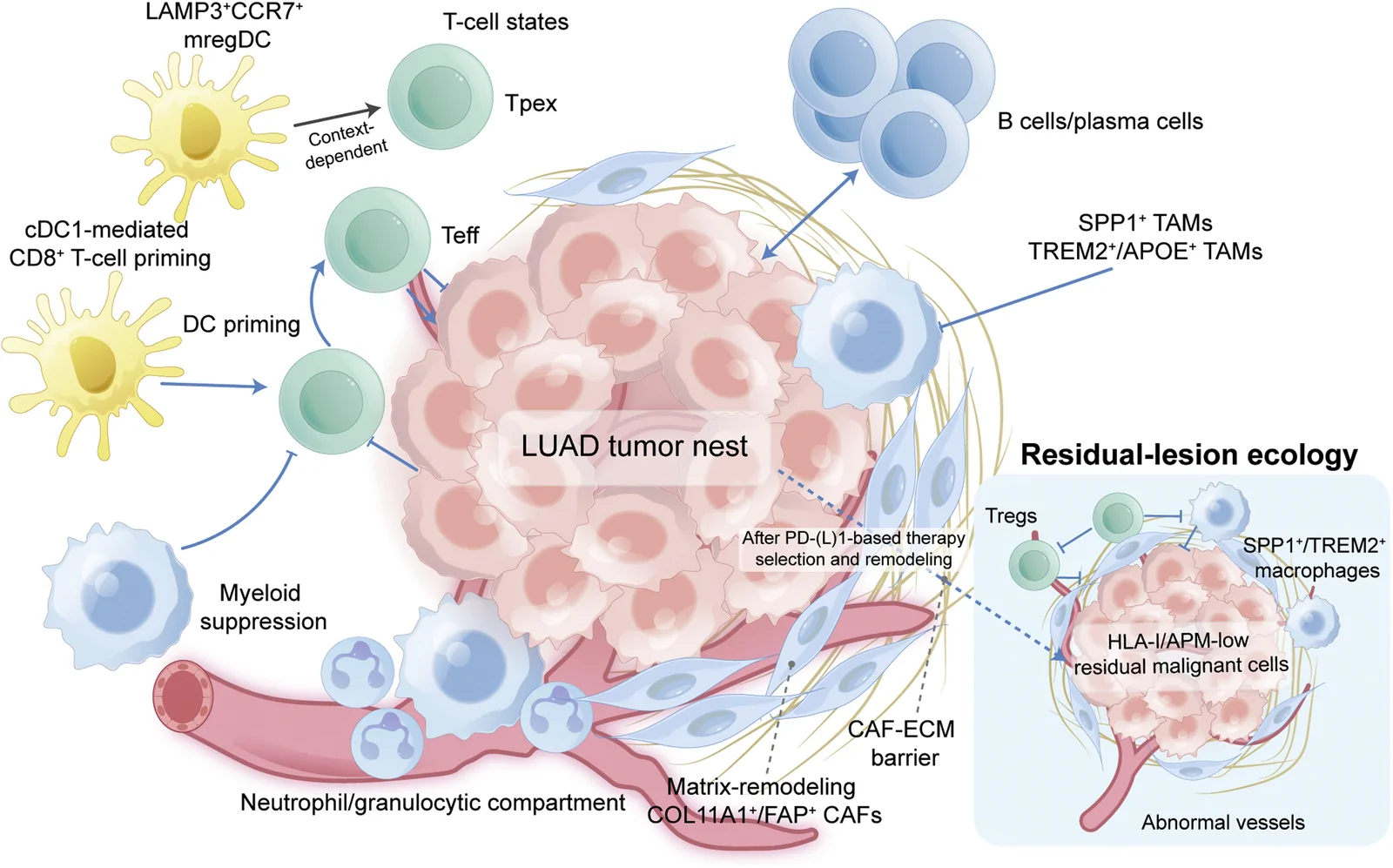
**Single-cell-defined immune-stromal remodeling and resistant community formation.** The schematic summarizes how LUAD tumor nests reshape surrounding immune and stromal compartments during or after PD-(L)1-based therapy. cDC1-mediated antigen presentation supports CD8+ T-cell priming, whereas LAMP3+CCR7+ mature/regulatory dendritic-cell states may coordinate immune activation or contribute to context-dependent immune regulation. T-cell compartments include precursor exhausted T cells, effector T cells, terminally exhausted states, and regulatory T cells; therapeutic response depends not only on total T-cell infiltration, but also on tumor-reactive clonal expansion, reactivation potential, and physical access to tumor nests. B cells, plasma cells, and TLS-related programs may support local immune organization when spatially connected to antigen-rich tumor regions. Resistance is reinforced by suppressive myeloid states, including SPP1+ and TREM2+/APOE+ TAMs, neutrophil or granulocytic compartments, and matrix-remodeling COL11A1+/FAP+ CAFs that generate CAF-ECM barriers and limit T-cell penetration. The inset depicts residual-lesion ecology after PD-(L)1-based therapy, in which antigen-presentation/APM-low residual malignant cells coexist with Tregs, suppressive macrophages, abnormal vessels, and protective stroma, potentially forming a seedbed for acquired resistance and recurrence. Abbreviations: APM, antigen-processing machinery; APOE, apolipoprotein E; BCR, B-cell receptor; CAF, cancer-associated fibroblast; cDC1, type 1 conventional dendritic cell; CCR7, C—C chemokine receptor 7; CD8, cluster of differentiation 8; COL11A1, collagen type XI alpha 1 chain; DC, dendritic cell; ECM, extracellular matrix; FAP, fibroblast activation protein; HLA-I, human leukocyte antigen class I; LAMP3, lysosomal-associated membrane protein 3; LUAD, lung adenocarcinoma; mregDC, mature regulatory dendritic cell; PD-(L)1, programmed cell death protein 1/programmed death-ligand 1; SPP1, secreted phosphoprotein 1; TAM, tumor-associated macrophage; TCR, T-cell receptor; Teff, effector T cell; TLS, tertiary lymphoid structure; Tpex, precursor/progenitor exhausted T cell; Treg, regulatory T cell; TREM2, triggering receptor expressed on myeloid cells 2.

Thus, immune and stromal remodeling forms the second layer of the causal chain of immunotherapy resistance in LUAD. Malignant cells reduce visibility and release remodeling signals. DCs, T cells, B cells/TLSs, myeloid cells, CAFs, the ECM, and vascular ecosystems then undergo coordinated reorganization. Reversible inflammatory responses may bring benefit from ICIs, whereas myeloid-CAF-vascular barriers and protective structures in residual lesions promote stabilization of resistance. Single-cell transcriptomics reveals these cell states; TCR/BCR sequencing tracks clonal fate; and spatial omics further explains how these states combine within tissues into spatial immune niches such as immune-inflamed, immune-excluded, myeloid-rich, antigen-presentation-low residual, and TLS-associated niches [16,19,41]. Once malignant epithelial programs are established, resistance becomes durable only when these signals are received, amplified, and spatially organized by immune, stromal, and vascular cells. Single-cell transcriptomics, complemented by TCR/BCR profiling and spatial validation, resolves how dendritic-cell priming, T-cell clonal fate, B-cell/plasma-cell responses, myeloid suppression, CAF/ECM barriers, granulocytic compartments, and residual-lesion ecology interact around LUAD tumor nests (Fig. 3).

## Spatial immune niches: tissue units linking mechanisms, pathologic architecture, and resistance phenotypes

The core contribution of spatial omics is to translate cellular composition into tissue structure. The same number of CD8+ T cells, located in the tumor nest, CAF-rich stroma septum, around TLS or necrotic margin, have completely different biological significance [41]. The same SPP1+ macrophages may have different communication targets and functional consequences if they are distributed in hypoxic areas, perivascular areas, or invasive fronts [45,82]. To facilitate mechanism stratification, the local spatial immune niche could be summarized into five categories. Real lesions more often show the coexistence, transition and nesting of multiple types of niches.

### T cell-inflamed niches with adaptive suppression

These regions usually have relatively complete antigen presentation, spatial proximity between CD8+ T cells and antigen presenting cells/malignant cells, IFN response and CXCL9/10 inflammatory chemotactic signals, suggesting the presence of immune responses that can be released by ICI in the tumor. Drug resistance often comes from PD-L1, IDO1, CTLA-4, TIGIT, LAG3, TIM-3 and other adaptive inhibitory programs counteracting T cell effects [15,22,26]. The assessment should focus on whether progenitor exhausted T cells are retained, whether TCR clones are expanded, whether antigen presentation is intact, and whether inhibitory signals are located at the tumor-T-cell contact interface and are reversible.

### Immune-Excluded stromal-boundary niches

In such areas, T cells are present in the tissue but are confined to the tumor margin, fibrotic stroma, or blood vessels, making it difficult to penetrate the tumor nest [15,27]. CAF/ECM, TGF-β, CXCL12, abnormal blood vessels, EMT-like malignant cells, and myeloid cells together constitute a repulsive boundary. Cases showing T-cell infiltration by conventional immunohistochemistry may still be resistant to PD-1/PD-L1 blockade, often because of the long distance between T cells and tumor cells, the low probability of contact, and the blocking of the migration path by the stroma [85]. Spatial metrics such as the proportion of T cells within the tumor-stroma partition, T cell infiltration distance, CAF/ECM barrier thickness, and tumor-T cell contact score are closer to the functional nature of this niche than CD8 density alone.

### Myeloid-rich hypoxic-metabolic suppressive niches

Such areas are often located around necrosis, deep hypoperfusion, or near abnormal blood vessels and are enriched for programs related to hypoxia, glycolysis, lactate/adenosine, VEGFA, SPP1+ or TREM2+/APOE+ macrophages, monocytes, and neutrophils [46,86,87]. The mechanism is that hypoxic metabolic stress, abnormal blood vessels, and myeloid reprogramming form a closed loop: hypoxia promotes the accumulation of VEGF and metabolites, abnormal blood vessels restrict T cell entry, and myeloid cells further inhibit effector T cells and promote ECM remodeling. This niche is often associated with primary resistance or residual after treatment. A considerable part of the existing evidence still comes from correlation analysis [46,88]. Targeting SPP1, TREM2, or the adenosine axis as a therapeutic target requires demonstration of spatial localization, receptor cells, functional consequences, and drug perturbation effects.

### Antigen-presentation-low residual niches

After immunotherapy or chemoimmunotherapy, sensitive tumor cells are eliminated, and residual cells may be enriched for low HLA-I, stress tolerance, EMT-like, cell cycle arrest, hypoxic metabolism, or stem-like programs [58,89,90]. These residual cells are often protected by CAF, myeloid cells, or areas of low perfusion, forming a spatial soil for acquired resistance and relapse. Surgical specimens after neoadjuvant therapy are especially suitable for studying this niche because paired comparisons between pretreatment biopsies and post-treatment residual lesions can distinguish pre-existing resistant seeds from therapy-induced states [91].

### TLS-Associated immune-organized niches

In this review, we use ‘mature TLS’ to denote structures with well‑defined B‑cell follicles, T‑cell zones, follicular dendritic cells, and HEV‑like vessels, as assessed by histology or multiplex imaging. A ‘functional’ TLS additionally requires: (i) spatial proximity to tumor antigen‑rich regions (e.g., within 200 µm or in direct contact with tumor nests), (ii) evidence of plasma‑cell differentiation and BCR clonal maturation, (iii) presence of Tfh‑like CD4⁺ T cells and LAMP3⁺/CCR7⁺ dendritic cells, and (iv) demonstrable T‑cell egress from the TLS towards tumor nests, reflected by CD8⁺ T‑cell gradients and spatial contact with malignant cells. These criteria, though provisional, provide a framework to distinguish TLSs that are merely present from those that are actively contributing to antitumor immunity.

Mature TLSs represent relatively organized local immune responses, but a TLS-associated immune-organized niche should not be equated simply with immunotherapy sensitivity. TLSs located near tumor antigen regions and accompanied by cDCs, Tfh-like cells, BCR clonal maturation, plasma-cell differentiation, and tumor-reactive T/B clones may support sustained antitumor immunity [[92], [93], [94]]. TLSs that are distant from tumor nests, structurally immature, or surrounded by Tregs, suppressive myeloid cells, CAF/ECM barriers, and hypoxic regions may be bystander-like inflammatory structures, or may be unable to convert local immune priming into intratumoral killing [95,96].

Peyraud et al. specifically addressed this boundary issue in Cell Reports Medicine in 2025^51^. The study confirmed that mature TLSs in NSCLC are generally associated with better ICI outcomes. However, spatial transcriptomics and multiplex immunofluorescence further showed that in mTLS-positive NSCLC, two CAF subsets can mediate primary resistance to ICIs. These CAF programs are associated with immune exclusion, CD8+ T-cell exhaustion, and regulatory CD4+ T-cell infiltration. This result refines the boundary conditions of TLS biomarkers: mature TLSs indicate the potential for local immune priming, while the surrounding CAF/ECM and suppressive myeloid structures determine whether this potential can be converted into killing within tumor nests. Thus, the presence of a morphologically mature TLS does not guarantee a ‘functional’ TLS as defined above; the latter requires not only proper structure but also spatial access to tumor antigen regions and effective T-cell egress, both of which can be blocked by surrounding suppressive CAF and myeloid programs. Therefore, assessment of TLS-associated niches should include TLS maturity, TLS-to-tumor-nest distance, CAF/ECM density surrounding TLSs, Treg/myeloid burden, and CD8+ T-cell infiltration distance, rather than measuring only CXCL13, CD20, or CD138.

### Niche coexistence, transition, and sampling error

Niche classification is a mechanistic abstraction, whereas clinical specimens commonly display mixed states. One side of a tumor nest may be a CAF-rich exclusionary boundary, another side may be a T cell-inflamed region, deeper areas may show hypoxic metabolism and myeloid enrichment, and TLSs may be present at the margins [27,97]. A core needle biopsy may pass through only a transition zone between two niches, or may completely miss the invasive front, TLSs, and perinecrotic regions [98]. In such settings, a label such as inflamed, excluded, or myeloid-rich derived from a single biopsy may only reflect a local section and may not represent the entire immune ecology of the lesion.

Using multiregion NSCLC analysis, Jia et al. showed in Nature Communications in 2018 that regional mutational diversity and intratumoral immune responses exhibit spatial heterogeneity, suggesting that use of a single-region TMB or neoantigen load to infer immunogenicity underestimates local differences [99]. Recent spatial studies of immunotherapy have advanced this issue from multiregion genomic differences to differences in spatial niches. In NSCLC specimens collected before PD-1 blockade, Chen et al. found that response-associated stem-immunity hubs have distinct spatial organization and are different from mature TLSs [50]. In multiple cohorts of advanced NSCLC treated with PD-1-based immunotherapy, Aung et al. constructed spatial proteomic and compartment-based transcriptomic signatures and showed that vascular, granulocytic, tumor-proliferative, and immune-cell compartments can jointly predict outcomes [41]. These findings indicate that spatial signatures must address the methodological challenge of how to infer the whole from the local. A single biopsy site may capture a T cell-inflamed region while missing a CAF-rich exclusionary boundary, a hypoxic myeloid region, or a TLS-associated niche. Conversely, it may sample necrotic or fibrotic tissue and underestimate regions that still retain immune activity.

For clinical translation, multiregion sampling, whole-slide scanning, multiplex immunofluorescence, or spatial protein panels can be used to capture niche mixtures. Spatial-omics findings can also be compressed into digital pathology, H&E image features, spatial colocalization of PD-L1 and immune cells, or radiomic models to estimate ecological states in unsampled regions [85,99]. Overconfidence must still be avoided. Strong performance of a spatial model in a discovery cohort does not mean that it will be stably reproduced in small biopsies, post-treatment necrotic specimens, or metastatic lesions.

Spatial niches can also explain metastatic lesions and mixed responses. Brain, bone, and adrenal metastases have organ-specific vascular barriers, resident immune cells, and metabolic environments [100,101]. The same clone may form different immune niches in different organs [102]. For example, a T cell-inflamed region in the primary lesion and a myeloid-rich boundary in a brain metastasis can coexist within the same patient, leading to radiographic mixed response or oligoprogression [103,104]. Integrating primary, residual, recurrent and metastatic lesions into the same spatial ecological framework can help to transform the interlesion heterogeneity into a mechanistic problem that can be studied.

**Fig. 4:**
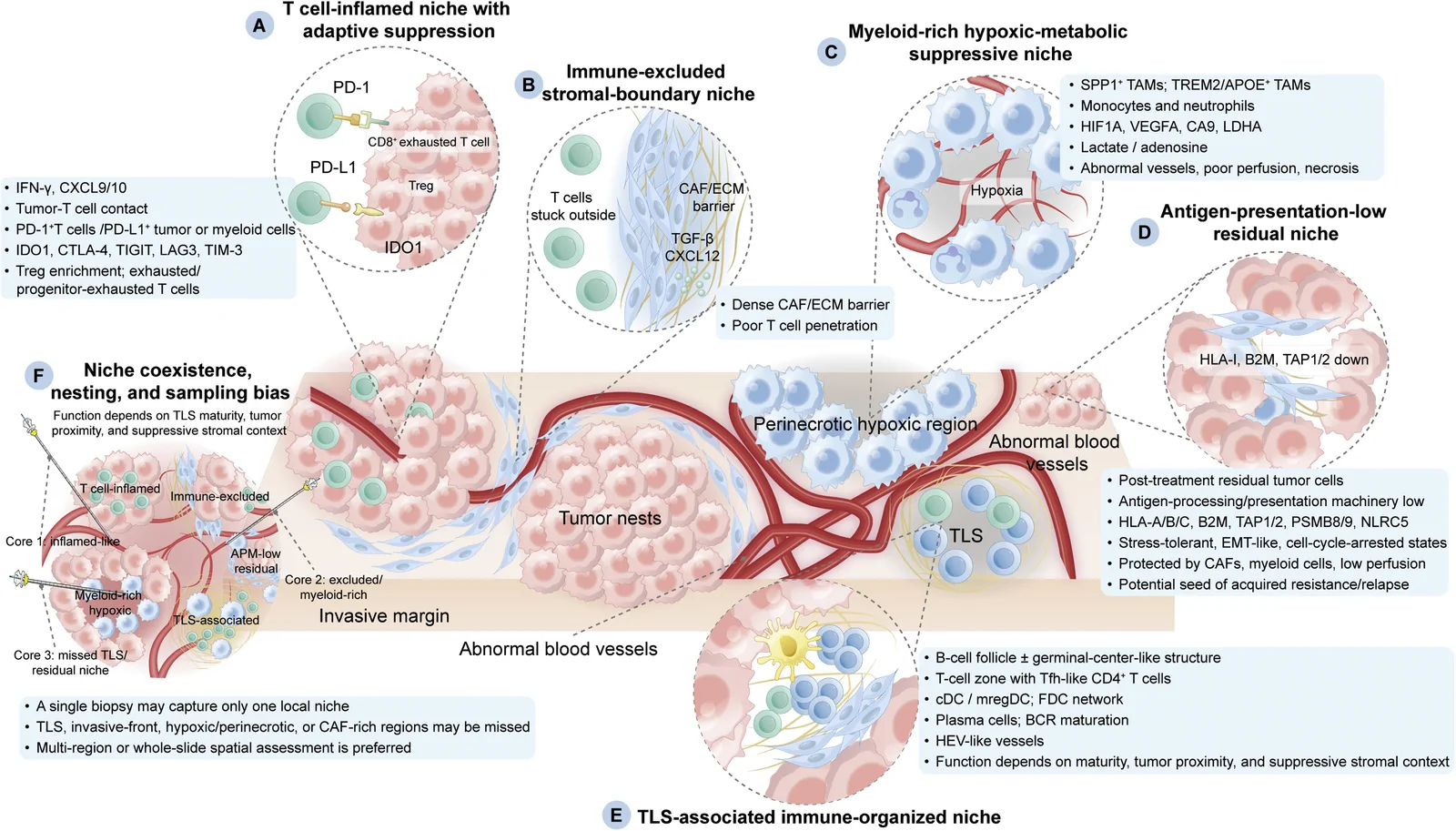
**Spatial immune niches linking tissue architecture to immunotherapy response and resistance.** The figure summarizes five major spatial immune niches that connect tissue architecture with immunotherapy resistance in LUAD/NSCLC. (A) T cell-inflamed niche with adaptive suppression: antigen presentation, IFN-γ/CXCL9/10 signaling, and tumor-T-cell contact indicate an ongoing immune response, but PD-1/PD-L1, IDO1, CTLA-4, TIGIT, LAG-3, TIM-3, Treg enrichment and terminally exhausted T-cell states may limit effective tumor killing, whereas retained precursor-exhausted T cells may indicate reactivatable antitumor immunity. (B) Immune-excluded stromal-boundary niche: T cells are present but remain outside tumor nests because dense CAF/ECM structures, TGF-β/CXCL12 programs, abnormal vasculature, and tumor-stromal interfaces impair T-cell penetration. (C) Myeloid-rich hypoxic-metabolic suppressive niche: poorly perfused or perinecrotic regions are enriched for HIF1A/VEGFA/CA9/LDHA, lactate and adenosine pathways, SPP1+ and TREM2+/APOE+ TAMs, monocytes/neutrophils, and abnormal vessels. (D) Antigen-presentation-low residual niche: after therapy, residual tumor cells may downregulate HLA-I, B2M, TAP1/2, and other antigen-processing machinery components while acquiring stress-tolerant, EMT-like, cell-cycle-arrested, or hypoxic-metabolic states protected by CAFs, myeloid cells, and low perfusion. (E) TLS-associated immune-organized niche: mature or functional TLSs contain B-cell follicle or germinal-center-like structures, T-cell zones with Tfh-like CD4+ T cells, cDC/mregDC populations, plasma cells, BCR maturation, and HEV-like vessels; their functional impact depends on TLS maturity, proximity to tumor antigen regions, and the surrounding suppressive stromal context. (F) A single biopsy may capture only one local niche. TLSs, invasive fronts, hypoxic/perinecrotic regions, or CAF-rich regions may be missed. Multi-region or whole-slide spatial assessment is preferred. Abbreviations: APM, antigen-processing machinery; APOE, apolipoprotein E; B2M, beta-2-microglobulin; BCR, B-cell receptor; CA9, carbonic anhydrase 9; CAF, cancer-associated fibroblast; cDC, conventional dendritic cell; CD4/CD8, cluster of differentiation 4/8; CTLA-4, cytotoxic T-lymphocyte-associated protein 4; CXCL9/10, C-X-C motif chemokine ligand 9/10; CXCL12, C-X-C motif chemokine ligand 12; ECM, extracellular matrix; EMT, epithelial-mesenchymal transition; FDC, follicular dendritic cell; HEV, high endothelial venule; HIF1A, hypoxia-inducible factor 1 subunit alpha; HLA, human leukocyte antigen; HLA-I, human leukocyte antigen class I; IDO1, indoleamine 2,3-dioxygenase 1; IFN, interferon; LAG3, lymphocyte activation gene 3; LDHA, lactate dehydrogenase A; LUAD, lung adenocarcinoma; mregDC, mature regulatory dendritic cell; NLRC5, NLR family CARD domain-containing 5; NSCLC, non-small cell lung cancer; PD-1, programmed cell death protein 1; PD-L1, programmed death-ligand 1; PSMB8/9, proteasome subunit beta 8/9; SPP1, secreted phosphoprotein 1; TAM, tumor-associated macrophage; TAP1/2, transporter associated with antigen processing 1/2; Tfh, follicular helper T cell; TGF-β, transforming growth factor beta; TIGIT, T-cell immunoreceptor with Ig and ITIM domains; TIM-3, T-cell immunoglobulin and mucin-domain containing-3; TLS, tertiary lymphoid structure; Treg, regulatory T cell; TREM2, triggering receptor expressed on myeloid cells 2; VEGFA, vascular endothelial growth factor A.

It is important to emphasize that ligand-receptor analysis and spatial colocalization can only suggest mechanistic hypotheses. Transcriptional coexpression does not equate to protein interaction, and spatial proximity does not equate to communication occurring. A reliable spatial niche needs to be verified by multiplex immunofluorescence, RNAscope, spatial proteome, in situ protein detection, tumor slice culture, organoid immune cell co-culture, animal model and drug/CRISPR perturbation. The goal of spatial omics should not stop at finding certain cells in proximity, but rather identify local tissue units that truly determine drug resistance and can be clinically detected and interfered with. At the tissue level, these cellular programs are best interpreted as spatial immune niches rather than isolated cell populations or marker lists. The same cell type or molecular signal may indicate response or resistance depending on its topographic relationship to tumor nests, stromal septa, hypoxic or perinecrotic regions, abnormal vasculature, and TLSs. The major niche patterns and the problem of niche coexistence and sampling bias are summarized in Fig. 4.

## From spatial ecological atlases to precision immune modulation: composite biomarkers and mechanism-matched strategies

If lung adenocarcinoma immunotherapy resistance is a spatial organized ecological process, biomarkers also need to be upgraded from a single molecule or a single cell proportion to a composite spatial ecological signature. A more reasonable model would simultaneously assess malignant cell immune visibility, T cell reversibility, antigen-presenting cell support capacity, myeloid inhibitory burden, CAF/ECM rejection structures, hypoxic metabolic stress, TLS maturity, and tumor-immune cell spatial contact. Such a signature should avoid stacking all the markers, and instead put each indicator in a clear causal position.

Malignant cell-level signatures should answer three questions: Can the tumor be recognized? Is it susceptible to killing? Does it actively shape a suppressive environment? The HLA-I/B2M/TAP/PSMB/NLRC5 module can assess antigen presentation [15,56]. IFN-response and PD-L1/IDO1 modules can identify adaptive suppression. EMT/TGF-β and hypoxia/glycolysis modules can indicate exclusion and metabolic tolerance [15,105]. Residual-cell-state scores can be used to assess recurrence risk after treatment [21,106]. Interpretation of these modules must incorporate spatial location. The same IFN-high signal may indicate reversible inflammatory suppression if it is located in tumor nests with sufficient T-cell contact [107]. If it is located in regions surrounded by myeloid cells and CAFs, it more closely resembles a tolerance signal after chronic stimulation.

T-cell-related signatures should include abundance, function, clonality, and spatial accessibility. Candidate dimensions include CD8+ T-cell abundance, cytotoxic score, progenitor exhausted T-cell score, terminal exhausted T-cell score, TCR clonal expansion, Tpex/Tex ratio, CXCL13+ tumor-reactive-like T-cell signature, and tumor-T cell contact score. Compared with total CD8 density, the presence of a progenitor exhausted T-cell pool, expansion of tumor-reactive clones, and entry of T cells into tumor nests are closer to the essence of whether ICIs can reactivate effective immunity.

TLS and B-cell signatures should be upgraded from the presence or absence of TLSs/B cells to assessment of maturity, functional state, and spatial accessibility. Candidate features include CXCL13/CCL19/CCL21, B-cell/plasma-cell modules, Tfh-like modules, DC modules, HEV-like endothelial signals, BCR clonal maturation, distance between TLSs and tumor nests, Treg/myeloid-cell burden around TLSs, and the influence of CAF/ECM on T-cell trafficking between TLSs and tumor nests. Mature TLSs located near tumor antigen regions may indicate stronger local immune-priming capacity. TLSs surrounded by hypoxia, Tregs, SPP1+ myeloid cells, or FAP+/α-SMA+ CAFs may be unable to convert local immune priming into intratumoral killing. A clinically translatable TLS/B-cell biomarker should therefore not be a single marker, but a composite score integrating TLS maturity, plasma-cell differentiation, tumor proximity, and the suppressive stromal context [48,49,51].

Myeloid, CAF/ECM, and hypoxic-metabolic signatures should be used to identify why an existing immune response fails to function, rather than simply to search for new single-gene targets. Candidate dimensions include CXCL9:SPP1 macrophage polarity, SPP1+/TREM2+/APOE+ macrophage burden, SPP1-CD44 or NECTIN2-TIGIT macrophage-T cell communication, the CCL2/CSF1 axis, neutrophil suppressive modules, CAF/ECM exclusion scores, TGF-β/CXCL12 modules, fibrosis/ECM-remodeling scores, HIF1A/VEGFA/CA9/LDHA/adenosine modules, and myeloid-T cell or CAF-T cell spatial distance. The most important pitfall is to treat a marker directly as a target [108]. A module is suitable for clinical stratification or combination-treatment design only if it is reproducible in independent cohorts, associated with outcome, spatially localized, and perturbable in functional models. In NSCLC, SPP1+ macrophages, FAP+/COL11A1+ CAFs, vascular/granulocytic compartments, and fibrosis scores are better integrated as spatial ecological indicators of immune-excluded or myeloid-rich hypoxic-metabolic niches than listed separately as independent biomarkers [41].

Therapeutic strategies should be matched to niche mechanisms. Antigen-presentation-low or immune-cold tumors require exploration of approaches that enhance immunogenicity, induce antigen presentation, promote immunogenic cell death through radiotherapy or chemotherapy, regulate epigenetic programs, or activate innate immunity. T cell-inflamed tumors with increased adaptive suppression may be better suited for investigation of CTLA-4, TIGIT, LAG3, or other immunomodulatory axes based on PD-1/PD-L1 blockade. Myeloid-rich hypoxic-metabolic tumors should be prioritized for strategies targeting myeloid recruitment, survival, or polarization; vascular normalization; hypoxia; and adenosine/lactate metabolic regulation. In CAF/ECM-rich or immune-excluded tumors, simply enhancing T-cell function is often insufficient. It is more important to relieve obstacles involving TGF-β, CXCL12/CXCR4, ECM remodeling, abnormal vasculature, and T-cell trafficking. TLS-mature immune-organized tumors require preservation of local antigen presentation, T/B-cell cooperation, and effector T-cell function, while avoiding indiscriminate disruption of beneficial immune structures.

Neoadjuvant and perioperative therapy are priority settings for validating these models. Compared with single biopsies in advanced disease, neoadjuvant therapy allows acquisition of pretreatment biopsies and post-treatment surgical specimens and enables spatial ecological states to be linked with MPR, pCR, residual viable tumor proportion, ctDNA clearance, event-free survival, and recurrence patterns. Post-treatment residual lesions directly display malignant cell states selected by immune pressure, T-cell clonal fate, myeloid/CAF protective structures, and potential recurrence niches. Perioperative studies should incorporate single-cell/spatial omics as prespecified translational endpoints and define sampling regions and outcome measures in advance.

The timing and sequence of mechanism‑matched combinations may be as critical as the choice of agents. Pre‑clinical and emerging clinical data suggest that a ‘priming’ phase – for example, using radiotherapy, chemotherapy, or epigenetic modulators to induce immunogenic cell death or upregulate antigen presentation – could render tumors more susceptible to subsequent immune checkpoint blockade. For myeloid‑rich or CAF‑excluded niches, concurrent or early administration of vascular normalizers or TGF‑β inhibitors might be needed to create physical access before T‑cell‑directed therapies are given. In TLS‑organized tumors, preserving local immune structures argues against early disruption; instead, sequential addition of agents that relieve per TLS suppressive barriers (e.g., Treg or myeloid modulators) after PD‑(L)1 blockade could be more rational. Although definitive evidence for optimal sequencing is still limited, the spatial ecological model encourages adaptive trial designs that incorporate serial biopsies and dynamic imaging to inform timing decisions.

Clinical translation must also face technical and design limitations. Single-cell sequencing has tissue dissociation bias, and fragile cells, stromal cells and space-dependent states may be lost. Spatial transcriptomes have resolution, spot mixing, deconvolution error, and platform batch effect. Single biopsy may miss immune-excluded boundaries, TLS or hypoxic regions. Many studies have limited sample sizes and mix different pathological types, stages, treatment lines, and sampling times [109,110]. LUAD-specific evidence and mixed NSCLC evidence need to be clearly distinguished. A more clinically realistic approach would be to first build spatial single-cell signatures in the discovery cohort and then compress them into FFPE-compatible multiplex immunofluorescence, RNAscope, spatial proteomics panel, targeted transcriptome panel, or digital pathology-transcriptome joint model. These results should be validated by independent cohorts, cross-platform reproducibility, prespecified thresholds, and prospective clinical trials Table 2.

**Table 2:** Composite spatial ecological biomarkers and mechanism-matched therapeutic strategies.

Spatial ecotype / niche	Composite biomarker pattern	Clinically compressible readout	Spatial interpretation	Mechanism-matched candidate strategy	Evidence context / caveat	Key references
T cell-inflamed niche with adaptive suppression	Preserved HLA-I/APM signals; IFN-γ/CXCL9/10 activity; CD8⁺ T-cell infiltration; Tpex or tumor-reactive-like CD8⁺ T cells; PD-L1, IDO1, CTLA-4, TIGIT, LAG-3, TIM-3, ENTPD1, and Treg-related programs	FFPE mIF or spatial proteomics for CD8, GZMB, PD-1, PD-L1, FOXP3, IDO1, LAG-3/TIGIT; targeted RNA panel for IFN-response and chemokine modules; spatial tumor–T-cell contact score; optional TCR clonality when tissue allows	An immune response is present and physically close to tumor nests, but tumor killing is restrained by adaptive inhibitory feedback or Treg-dominant suppression	Maintain PD-1/PD-L1 blockade as the conceptual backbone; rationally evaluate CTLA-4, TIGIT, LAG-3/TIM-3, IDO1, or adenosine-axis combinations only when the inhibitory program is spatially concentrated at tumor–immune interfaces	Response-permissive rather than response-guaranteeing. Total CD8 density is insufficient; T-cell clonality, Tpex retention, antigen presentation, and spatial access are required for interpretation	[19,22,26,42]
Antigen-presentation-low / immune-invisible niche	Low HLA-A/B/C, B2M, TAP1/2, PSMB8/9, NLRC5; HLA LOH or allele-specific HLA disruption; B2M/JAK1/JAK2 defects; weak pMHC-I–TCR recognition despite nearby immune cells	HLA-I and B2M IHC/mIF; RNAscope or targeted RNA panel for APM genes; DNA/RNA NGS for HLA LOH, B2M, JAK1/2 and IFN-pathway alterations; spatial comparison of CD8 proximity versus tumor HLA-I status	Tumor cells may be present in immune-infiltrated tissue but remain poorly visible to cytotoxic T cells; residual islands may escape through impaired antigen processing or IFN responsiveness	Explore immunogenicity-restoring approaches, including radiotherapy/chemotherapy-induced immunogenic stress, epigenetic priming, STING/TLR agonism, or innate/NK-cell-oriented strategies in appropriate trials; avoid assuming that additional checkpoint blockade alone will overcome fixed APM loss	Transcript-level APM reduction is not equivalent to functional antigen-presentation loss. Protein validation and genomic confirmation are needed before using this as a treatment-stratification feature	[11,29,43,89]
Immune-excluded CAF/ECM boundary niche	CD8⁺ T cells retained in stroma or invasive margin; ACTA2/TAGLN myCAF features; FAP, COL1A1, COL11A1, POSTN, LOX, MMP-related ECM remodeling; TGF-β/CXCL12 programs; partial EMT or AXL-related tumor states	H&E tumor–stroma partitioning; mIF/spatial proteomics for CD8, panCK, α-SMA, FAP, COL1A1/COL11A1, POSTN; collagen staining or second-harmonic imaging where available; T-cell infiltration distance, CAF/ECM barrier thickness, and tumor–T-cell contact score	T cells are present in the specimen but are physically separated from tumor nests by fibrotic or matrix-rich stromal barriers; this may create a pseudo-inflamed but functionally excluded phenotype	Evaluate combinations that relieve physical or chemotactic exclusion, such as TGF-β-axis modulation, CXCL12/CXCR4 blockade, ECM/fibrosis remodeling, LOX-related matrix modulation, or vascular normalization, preferably with PD-1/PD-L1 blockade rather than instead of it	CAFs are heterogeneous; not all CAF-rich states are suppressive. Broad CAF depletion may be biologically unsafe or counterproductive. Subset- and location-specific validation is required	[15,27,44,82]
Myeloid-rich hypoxic-metabolic suppressive niche	SPP1⁺, TREM2⁺/APOE⁺, or lipid-metabolic TAM states; S100A8/A9⁺ or CXCR2-related granulocytic/neutrophil modules; low CXCL9:SPP1 ratio; HIF1A, CA9, VEGFA, LDHA, lactate, adenosine/CD39/CD73-related programs	mIF/spatial proteomics for CD68/CD163, SPP1, TREM2, APOE, S100A8/A9, MPO, CD8, CA9, VEGFA, CD31; targeted RNA panel for CXCL9:SPP1, hypoxia, VEGF and adenosine modules; myeloid–T-cell distance and perinecrotic/perivascular localization	Hypoxia, abnormal perfusion, suppressive myeloid polarization, and metabolic competition form a local feedback loop that limits T-cell entry and effector function	Consider myeloid recruitment/survival/polarization strategies, vascular normalization, anti-VEGF combinations, hypoxia modulation, and adenosine/lactate-axis targeting as mechanism-matched hypotheses; TREM2- *O. spp1*-related interventions require stronger functional validation	SPP1 and TREM2 may be markers, mediators, or context-dependent consequences of hypoxic/stromal remodeling. Spatial co-localization and ligand–receptor inference alone are insufficient proof of targetability	[41,45,86,110]
TLS-associated immune-organized niche	Mature TLS with B-cell follicles, T-cell zones, FDC/HEV-like structures, CXCL13/CCL19/CCL21, LAMP3⁺/CCR7⁺ DCs, Tfh-like CD4⁺ T cells, plasma cells, and BCR maturation; surrounding Treg, myeloid, CAF or hypoxic barriers should be annotated	H&E TLS maturity assessment; mIF for CD20/CD79A, CD3, CD4, CD8, CD21/CD23, CD138, PNAd/HEV, LAMP3, FOXP3, FAP/α-SMA; TLS-to-tumor distance; CD8 trafficking from TLS to tumor nests; plasma-cell and BCR-related targeted RNA signatures	TLSs indicate local immune organization and potential antigen priming, but their functional value depends on maturity, tumor proximity, effector-cell egress, and the suppressive structures around them	Preserve or enhance productive local immune organization; support antigen presentation and T/B-cell cooperation; in mTLS-positive but resistant tumors, prioritize targeting surrounding CAF/ECM, Treg or myeloid barriers rather than treating TLS presence itself as sufficient	Mature TLS is generally response-permissive, but not deterministic. mTLS-positive primary resistance can occur when stromal or myeloid barriers prevent intratumoral killing	[[48], [49], [50], [51]]
Post-treatment residual / recurrence-seeding niche	Residual viable tumor with APM-low, IFN-altered, EMT/stress-tolerant, hypoxic-metabolic, cell-cycle-arrested or stem-like states; insufficient Tpex/Tex-relevant clonal expansion; CCR8⁺ Treg enrichment; SPP1⁺/TREM2⁺ myeloid cells; COL11A1⁺/FAP⁺ CAFs; persistent ctDNA	Paired pretreatment biopsy and post-treatment surgical specimen; residual viable tumor mapping; mIF/RNAscope around residual tumor islands; targeted RNA panel for APM, T-cell, Treg, myeloid, CAF and hypoxia modules; TCR/BCR tracking when feasible; ctDNA clearance and recurrence monitoring	Residual disease is not only “tumor left behind” but a selected tissue ecology in which resistant malignant states, impaired clonal immune control, suppressive stromal/myeloid structures, and local immune organization determine recurrence risk	Use perioperative adaptive designs to test mechanism-matched consolidation: continued PD-1-based therapy when reactivatable T-cell ecology persists; immunogenicity-restoring, myeloid/CAF-targeting, vascular or innate-immune approaches when residual niches are APM-low, excluded, or myeloid-hypoxic	Best suited for neoadjuvant and perioperative validation. Thresholds for residual ecological signatures are not standardized and require prospective outcome-linked validation	[11,12,21,71]

**Note**: This table is intended as a translational framework rather than a treatment guideline. Candidate strategies are hypothesis-generating and should be interpreted as translational-research or clinical-trial design directions, not as established treatment recommendations. Each spatial ecotype should be interpreted in relation to treatment regimen, sampling time point, lesion site, tissue quality, and platform resolution. Composite biomarkers require protein-level or spatial validation, reproducibility across cohorts, and prospective association with clinical outcomes before clinical implementation. Gene and protein marker symbols follow standard nomenclature and are not expanded individually.

**Abbreviations**: APM, antigen-processing machinery; BCR, B-cell receptor; CAF, cancer-associated fibroblast; DC, dendritic cell; ECM, extracellular matrix; EMT, epithelial–mesenchymal transition; FDC, follicular dendritic cell; FFPE, formalin-fixed paraffin-embedded; HEV, high endothelial venule; ICI, immune checkpoint inhibitor; IFN, interferon; LUAD, lung adenocarcinoma; mIF, multiplex immunofluorescence; NSCLC, non-small cell lung cancer; pMHC-I, peptide–major histocompatibility complex class I; TAM, tumor-associated macrophage; TCR, T-cell receptor; TLS, tertiary lymphoid structure; Tpex, precursor/progenitor exhausted T cell; Treg, regulatory T cell.

## Conclusions and perspectives

Immunotherapy resistance in NSCLC, with LUAD as a major clinical and molecular subtype, should be understood as a dynamic evolution and spatial organization of tumor immune ecological process. The molecular background and clinical scenario before treatment set initial conditions, treatment stress induces or selects malignant cells for state transition, and malignant cells remodel immunity and matrix through antigen presentation, IFN response, EMT/stress/metabolic adaptation and secretion programs. T cells, DC, B cells/TLSs, myeloid cells, CAF/ECM, and vascular ecology further amplify local escape into stable resistant structures. Ultimately, therapeutic efficacy is determined by how these cellular states are organized in tissue space into functional niches, including T-cell-inflamed/adaptively suppressed niches, immune-excluded stromal-boundary niches, myeloid-rich hypoxic-metabolic niches, antigen-presentation-low residual niches, and TLS-associated immune-organized niches, and by how these niches coexist and transition within and across lesions.

The significance of this framework is to advance high-resolution omics discovery from descriptive mapping to mechanistic stratification. Single-cell sequencing answers which cells are in which states, spatial omics answers where these states are located and interact with whom, and longitudinal sampling and functional perturbation answers which states truly drive resistance and can be interfered with. Future studies are needed to more strictly distinguish evidence of LUAD and mixed NSCLC, pre-treatment status and treatment-induced status, and correlation co-localization and functional causality. After spatial localization, protein validation, functional perturbation and clinical outcome association, the niche signature may become a reliable biomarker or basis for combination therapy.

From the translational perspective, the ultimate value of spatial single-cell research is to decompose complex tumor ecology into clinical units that can be detected, interpreted, and intervened. For patients with different ecotypes such as antigen-presentation-low, myeloid-rich, CAF/ECM-excluded, TLS-mature, or hypoxic-metabolic, mechanism-matched combination treatment strategies should be designed in the future. These strategies should be validated in perioperative and advanced-disease prospective studies. With the development of spatial-omics platforms, computational integration methods, and FFPE-compatible detection technologies, the study of immunotherapy resistance in lung adenocarcinoma is expected to enter the stage of spatial ecological diagnosis and precise immune regulation from the list of cell states. Future perioperative and adaptive-design trials should not only test the right combinations but also the right sequence, with serial tissue and imaging assessments to guide dynamic adjustments.

## Funding

The authors declare that no financial support was received for the research, authorship, and/or publication of this article.

## Data availability

Data availability is not applicable to this article as no new data were created or analyzed in this study.

## CRediT authorship contribution statement

**Yiming Hua:** Writing – original draft, Visualization. **Zhanyang Luo:** Validation. **Zhimei Huang:** Writing – review & editing, Supervision, Conceptualization.

## Declaration of competing interest

The authors declare that they have no known competing financial interests or personal relationships that could have appeared to influence the work reported in this paper.
